# Codon and Amino Acid Usage Are Shaped by Selection Across Divergent Model Organisms of the Pancrustacea

**DOI:** 10.1534/g3.115.021402

**Published:** 2015-09-17

**Authors:** Carrie A. Whittle, Cassandra G. Extavour

**Affiliations:** * Department of Organismic and Evolutionary Biology, Harvard University, Cambridge, Massachusetts 02138; † Department of Molecular and Cellular Biology, Harvard University, Cambridge, Massachusetts 02138

**Keywords:** *Gryllus bimaculatus*, *Oncopeltus fasciatus*, *Parhyale hawaiensis*, optimal codons, translational selection

## Abstract

In protein-coding genes, synonymous codon usage and amino acid composition correlate to expression in some eukaryotes, and may result from translational selection. Here, we studied large-scale RNA-seq data from three divergent arthropod models, including cricket (*Gryllus bimaculatus)*, milkweed bug (*Oncopeltus fasciatus*), and the amphipod crustacean *Parhyale hawaiensis*, and tested for optimization of codon and amino acid usage relative to expression level. We report strong signals of AT3 optimal codons (those favored in highly expressed genes) in *G. bimaculatus* and *O. fasciatus*, whereas weaker signs of GC3 optimal codons were found in *P. hawaiensis*, suggesting selection on codon usage in all three organisms. Further, in *G. bimaculatus* and *O. fasciatus*, high expression was associated with lowered frequency of amino acids with large size/complexity (S/C) scores in favor of those with intermediate S/C values; thus, selection may favor smaller amino acids while retaining those of moderate size for protein stability or conformation. In *P. hawaiensis*, highly transcribed genes had elevated frequency of amino acids with large and small S/C scores, suggesting a complex dynamic in this crustacean. In all species, the highly transcribed genes appeared to favor short proteins, high optimal codon usage, specific amino acids, and were preferentially involved in cell-cycling and protein synthesis. Together, based on examination of 1,680,067, 1,667,783, and 1,326,896 codon sites in *G. bimaculatus*, *O. fasciatus*, and *P. hawaiensis*, respectively, we conclude that translational selection shapes codon and amino acid usage in these three Pancrustacean arthropods.

The arthropods comprise more than 80% of animal species currently living and are a highly diverse phylum of exoskeleton organisms that includes terrestrial insects and aquatic crustaceans (Akam 2000; Odegaard 2000; Regier *et al.* 2010). Despite the diversity of this phylum, research in genome evolution in arthropods remains focused either on species of flies and mosquitoes, primarily *Drosophila* and *Anopheles* (Diptera) (Neafsey *et al.* 2015; Stark *et al.* 2007; Wiegmann and Yeates 2005), or on a few other holometabolous insects (those with complete metamorphosis; the so-called “higher” insects) such as beetles (Coleoptera) or wasps (Hymenoptera) (Brown *et al.* 2008; Group *et al.* 2010; Weinstock *et al.* 2006). Much remains unknown outside those insects, and a broader understanding of how arthropod genomes evolve requires expanding studies to include nontraditional models (Zuk *et al.* 2014). Emerging arthropod model systems for genomics research include two basally branching hemimetabolous insects, namely a cricket (*Gryllus bimaculatus*, Orthoptera) and the milkweed bug (*Oncopeltus fasciatus*, Hemiptera), as well as the amphipod crustacean *Parhyale hawaiensis* (Amphipoda) (Liu and Kaufman 2009a; Mito and Noji 2009; Rehm *et al.* 2008). Each of these arthropods has recently acquired genome-wide transcriptome data from ovaries, multistage embryos, and/or postembryonic tissues (Ewen-Campen *et al.* 2011; Zeng *et al.* 2011, 2013; Zeng and Extavour 2012), allowing investigation of evolutionary dynamics of protein-coding genes.

Crickets have historically been key systems for research in functional genetics, neurobiology, and developmental biology but have more recently emerged as an important model for evolutionary biology, including topics in sexual selection and speciation (Garcia-Gonzalez and Simmons 2010; Huber *et al.* 1989; Zuk *et al.* 2014). The two-spotted cricket *G. bimaculatus*, like other crickets, branches basally to the Diptera, Hymenoptera, and Coleoptera and has a large genome (∼1.7 Gb) predicted to contain more than 19,000 unique gene sequences (Zeng *et al.* 2013). An additional basally branching hemimetabolous insect, the milkweed bug *O. fasciatus*, provides further opportunities for molecular evolutionary research (Butt 1949; Liu and Kaufman 2009a,b). Specifically, more than 10,000 unique genes, including those involved in an array of developmental, signaling, and housekeeping functions, have been identified, allowing comparative genomics in this taxon (Ewen-Campen *et al.* 2011). The amphipod crustacean *P. hawaiensis* belongs to the crustacean class Malacostraca, which is one of the crustacean groups most closely related to the Hexapods (Insecta, Collembola, Protura, and Diplura) (Regier *et al.* 2010) and is common in intertidal habitats worldwide. It has extensive genetic and developmental tools available (Kontarakis *et al.* 2011; Pavlopoulos and Averof 2005; Rehm *et al.* 2008), yet remarkably is one of the only crustaceans besides *Daphnia pulex*, with whole genome or transcriptome datasets available (Colbourne *et al.* 2011; Zeng and Extavour 2012; Zeng *et al.* 2011). Currently, few studies are available regarding the genomic traits and evolution in nontraditional arthropod models, including the evolution of synonymous codon usage and amino acid composition in protein-coding DNA.

Synonymous codons are not used randomly in the genome. Biases in synonymous codon usage may arise from mutational pressure (Osawa *et al.* 1988; Sharp *et al.* 1995; Sueoka 1988) or from selective forces favoring efficient and accurate translation (Duret 2000; Duret and Mouchiroud 1999; Stoletzki and Eyre-Walker 2007). Findings that codon usage biases are correlated with transfer RNA (tRNA) abundance and/or gene copy number (Duret 2000; Ikemura 1981; Ikemura 1985) and with gene expression levels in numerous taxonomic groups (*e.g.*, *Escherichia coli*, *Saccharomyces*, *Caenorhabditis*, *Drosophila*, *Arabidopsis*, *Silene*, *Populus*) support the theory that selection favors specific codons that promote efficient and accurate translation of genes that are expressed at high levels (Cutter *et al.* 2006; Duret and Mouchiroud 1999; Ingvarsson 2008; Qiu *et al.* 2011; Sharp and Li 1986; Sharp *et al.* 1986). Thus, an effective method to identify codons favored by selection (optimal codons) is to compare codon usage per amino acid in highly expressed genes and lowly expressed genes (Cutter *et al.* 2006; Duret and Mouchiroud 1999; Ingvarsson 2008; Wang *et al.* 2011; Whittle *et al.* 2007). In this regard, the presence/absence of optimal codons and factors linked to their usage may reveal whether selection for translational efficiency and/or accuracy plays a major role in an organism’s genome evolution.

In arthropods, nearly all information to date about optimal codons is from higher insects: optimal codons have been identified in species of *Drosophila* and *Anopheles*, and other taxa such as *Tribolium castaneum*, *Apis mellifera*, and *Nasonia vitripennis* (Behura and Severson 2012, 2013; Duret and Mouchiroud 1999; Williford and Demuth 2012; C. A. Whittle *et al.*, unpublished data), although no or weak genome-wide trends were evident in *Bombyx mori* (Jia *et al.* 2015; C. A. Whittle *et al.*, unpublished data). The bias favors GC3 codons in some organisms, including *D. melanogaster* (Duret and Mouchiroud 1999), but AT3 is favored in others (Behura and Severson 2012; C. A.Whittle *et al.*, unpublished data), indicating marked divergence in the presence/absence of optimal codons and types of optimal codons among these insects. Nevertheless, further data are needed about the dynamics of codon usage in arthropods outside of the higher insects, such as hemimetabolous insects and crustaceans, to answer a number of outstanding questions, including, for example whether optimal codons exist in these organisms, and if so, how they are related to traits such as amino acid composition (Akashi 2003; Cutter *et al.* 2006; Williford and Demuth 2012).

The amino acid composition of proteins also appears to change with increasing expression levels (Akashi 2003; Cutter *et al.* 2006; Duret 2000; Williford and Demuth 2012). Several reports have indicated that amino acid frequency in proteins encoded by highly expressed genes correlates to the most abundant tRNA in an organism, consistent with selection for speed and accuracy of translation (Akashi 2003; Duret 2000), Further, studies in some eukaryotes, including yeast, *T. castaneum*, and *Caenorhabditis elegans*, have indicated that amino acids in highly expressed genes tend to be less metabolically costly (Cutter *et al.* 2006; Raiford *et al.* 2008; Williford and Demuth 2012). For instance, highly transcribed genes contain fewer amino acids of large size and complexity (S/C scores; Dufton 1997), and favor low cost amino acids (Akashi 2003; Cutter *et al.* 2006; Williford and Demuth 2012). Thus, in those studies, abundant proteins, presumably encoded by highly transcribed genes, tended to be comprised of smaller less complex amino acids, which is not only predicted to minimize the biochemical energy costs of synthesis, but might also contribute to the stability of the protein structure and its conformation (Cutter *et al.* 2006; Dufton 1997; Williford and Demuth 2012). Expanding this molecular evolutionary research to non-traditional model organisms will help elucidate the breadth of this phenomenon and the role of evolution of amino acid preferences in promoting translational efficiency.

In the present investigation, we study optimal codon and amino acid usage based on recently available large-scale RNA-seq data from three emerging models of arthropods, *G. bimaculatus*, *O. fasciatus*, and *P. hawaiensis*. The RNA-seq data are available at the Assembled Searchable Giant Arthropod Read Database (*i.e.*, ASGARD) (Ewen-Campen *et al.* 2011; Zeng *et al.* 2011; Zeng and Extavour 2012; Zeng *et al.* 2013). Using these data, we provide strong evidence that codon usage and amino acid frequency has been optimized in highly expressed genes of each of these organisms, with the strongest signals observed in *G. bimaculatus* and *O. fasciatus*, and weaker, yet significant effects detected in *P. hawaiensis*. Further, using *D. melanogaster* as a reference, we show that highly expressed coding sequence (CDS) are shorter, consistent with translational selection, and are enriched for genes involved in cell-cycle processes and protein synthesis in all three organisms. Together, the results are consistent with a history of selection on synonymous codon usage and on amino acid frequency in highly transcribed genes to promote translational efficiency and accuracy across Pancrustacean genome evolution.

## Materials and Methods

### Transcriptomes Under Study

Here, we studied RNA-seq data from ASGARD derived from ovaries, multi-stage embryos and/or postembryonic tissues for *G. bimaculatus*, *O. fasciatus*, and *P. hawaiensis* (Supporting Information, Table S1) (Ewen-Campen *et al.* 2011; Zeng *et al.* 2011; Zeng and Extavour 2012; Zeng *et al.* 2013). Multistage embryos and ovaries comprise highly complex tissues, expressing a large component of genes in the genome (Li *et al.* 2014); thus, genes highly transcribed in these tissues are apt to be an effective tool to reveal genome-wide optimization of codon usage and amino acids in an organism (c.f. Subramanian and Kumar 2004). The next-generation sequencing data are described in Table S1. We identified all assembled transcripts (Zeng and Extavour 2012) without isoforms, to allow accurate mapping of reads to a specific single CDS to quantify expression. For this gene set, we extracted all coding regions with a start codon, and lacking any unknown sites or internal stop codons. To quantify expression levels, we mapped reads to the CDS and calculated reads per million (RPM) using the non-normalized libraries that comprise the largest (or only) dataset per species (Table S1; where read number directly reflects abundance; average read length was 349, 297, and 400 bp per species, respectively) (Zeng and Extavour 2012). RPM was defined as the number of read matches to a CDS/Total number of reads matching all CDS × 1,000,000; see *Identification of optimal codons* for reads per kilobase million, RPKM). Tools and software for measuring expression and codon and amino acid usage in the various datasets are described in File S3.

### Data availability

Data are available at ASGARD (Ewen-Campen *et al*. 2011; Zeng *et al*. 2011; Zeng and Extavour 2012; Zeng *et al*. 2013). Details are provided in Table S1.

## Results

For our analyses, the number of CDS examined after excluding genes with isoforms, unknown sites, or internal stop codons for *G. bimaculatus*, *O. fasciatus*, and *P. hawaiensis* was 5284, 6161, and 6731, which spanned 1,680,067, 1,667,783, and 1,326,896 codons, respectively. The nucleotide composition varied across all CDS among organisms: it was AT-rich for *G. bimaculatus* (AT = 0.6177 ± 0.006; with similar levels of A and T) and *O. fasciatus* (0.6520 ± 0.006; similar levels of A and T) and AT was mildly higher than GC content in *P. hawaiensis* (0.5529 ± 0.0417).

**Figure 1:**
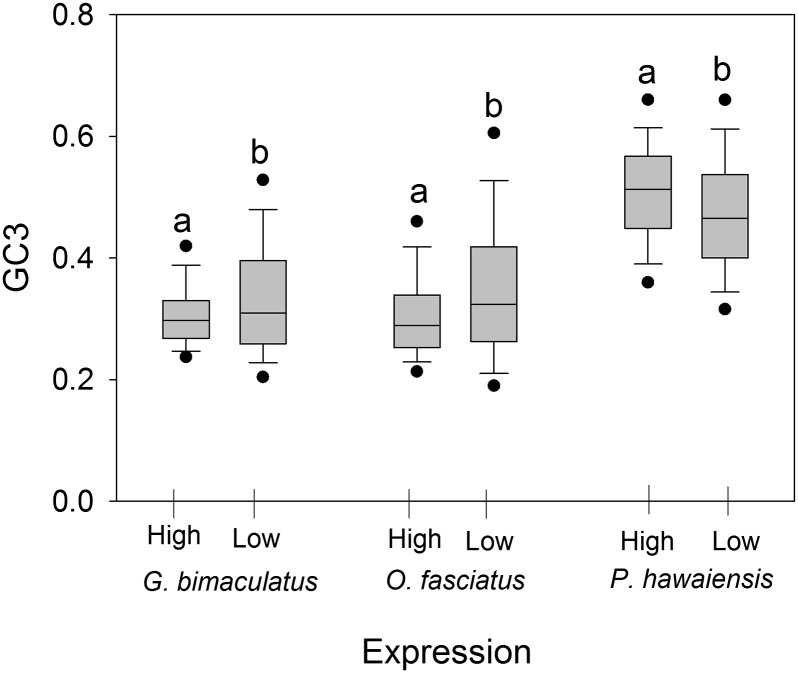
The GC3 content for the 5% most highly and lowly expressed genes for each of three species under study. Different letters indicate a statistically significant difference among high and low expressed genes within each species (*P* < 0.05 using *t*-tests).

We first asked whether we could detect differences in codon usage relative to expression levels in all three study species. To study synonymous codon usage, we first determined the GC3 content among the 5% highest and lowest expressed CDS and detected striking differences consistent with selection on codon usage among these two categories. A highly effective method to identify codon preferences is to compare these preferences between genes expressed at extremely high or extremely low levels (Cutter *et al.* 2006; Duret and Mouchiroud 1999; Ingvarsson 2008; Wang *et al.* 2011; Whittle *et al.* 2007, 2011a). As shown in Figure 1, we found that GC3 was statistically significantly lower in CDS with the 5% highest RPM (mean = 0.307 ± 0.003) than in the 5% of CDS with the lowest RPM (mean = 0.334 ± 0.006) in *G. bimaculatus* (*t*-test *P* = 2.0 × 10^−4^) and *O. fasciatus* (mean 5% highest = 0.306 ± 0.004 and mean 5% lowest= 0.349 ± 0.007, respectively, *P* = 1.5 × 10^−7^). These trends indicate that AT3 codons are more common under high transcription in these insects. For *P. hawaiensis*, GC3 was significantly elevated in highly expressed genes compared with lowly expressed genes (mean 5% highest = 0.507 ± 0.005 and mean 5% lowest = 0.473 ± 0.005 respectively, *P* = 6.8 × 10^−7^) suggesting selection for GC3 codons in highly expressed genes in this crustacean. For all genes per species, we then assessed the effective number of codons (ENC), wherein values range from 20 (when one codon is exclusively used to code for a given amino acid) to 61 (all codons used equally), and lower values denote greater biases in codon usage in a gene (Wright 1990). We found that AT3 was strongly negatively correlated with ENC in *G. bimaculatus* (Spearman’s Rank R = −0.65, *P* = 2.0 × 10^−6^) and *O. fasciatus* (R = −0.68, *P* = 2.0 × 10^−7^), thus concurring with AT3 codon preferences in those species. In contrast, no correlation was evident for GC3 and ENC across all genes from *P. hawaiensis*. (*P* > 0.05), but a negative correlation evident in the uppermost expressed gene set (R = −0.20, *P* < 2.0 × 10^−4^, for the uppermost 5%), implying marked GC3 favoritism within this dataset. Given these findings, we next assessed synonymous codon usage in every amino acid of the analyzed CDS, to test the hypothesis that AT3 in the insects, and GC3 in the amphipod, were optimal codons in these species.

### Identification of optimal codons

Although there is some variation in approaches to identify optimal codons (Hershberg and Petrov 2009, 2012; Wang *et al.* 2011), here we identified the optimal codon list in each amino acid in *G. bimaculatus*, *O. fasciatus*, and *P. hawaiensis* using relative synonymous codon usage (RSCU) relative to gene expression (Cutter *et al.* 2006; Duret and Mouchiroud 1999; Ingvarsson 2008; Qiu *et al.* 2011; Wang *et al.* 2011; Whittle *et al.* 2007; Whittle *et al.* 2011a). RSCU measures the observed frequency of a particular codon relative to the expected frequency if all synonymous codons were used equally. RSCU values greater than 1 indicate preferential usage, and higher values among codons within a synonymous codon family denote increased usage (Sharp and Li 1986). Optimal codons were defined as those having a statistically significant and positive ∆RSCU = RSCU_Mean Highly Expressed CDS_ − RSCU_Mean Low Expressed CDS_; when more than one codon matched this criterion per amino acid, the codon with the largest ∆RSCU amino acid was defined as the primary optimal codon (Cutter *et al.* 2006; Duret and Mouchiroud 1999; Ingvarsson 2008; Whittle *et al.* 2011a). The ∆RSCU for all amino acids is shown in Table 1, and the mean RSCU and standard errors per expression class are shown in Table S2. We report that 17 of the 18 amino acids with synonymous codons had an optimal codon with a statistically significant and positive ∆RSCU in *G. bimaculatus*. For *O. fasciatus*, we identified a total of 16 optimal codons. Both species favored AT3 codons in highly expressed genes. For instance, in *G. bimaculatus*, 15 of 17 optimal codons ended in AT, whereas 16 of 16 optimal codons ended in AT for *O. fasciatus*. In fact, for each of the eight amino acids encoded by four or more codons (*e.g.*, Ala, Arg, Gly, Leu, Pro, Ser, Thr, and Val), the optimal codon always ended in T in *O. fasciatus*, whereas six of eight (the two exceptions being Leu and Pro) ended in T for *G. bimaculatus*. Thus, there are marked preferences toward specific types of codons (T3) even for amino acids wherein an assortment of synonymous codons exist. The bias toward optimal codons ending in AT was also observed for amino acids with two or three synonymous codons (Table 1). As *G. bimaculatus* and *O. fasciatus* each have inherently AT rich transcriptomes (see first paragraph of the Results section), codon usage is likely partly influenced by the inherent genome composition, which might be expected to contain AT-rich codons. Despite this, our data show that the usage of AT3 codons is enhanced markedly in highly expressed genes (not expected under mutational pressure; see also *Optimal codon usage is shaped by selection*), and thus is consistent with selection favoring these optimal codons in genes that experience high rates of transcription.

**Table 1:** The difference (Δ) in mean RSCU for the 5% most highly *vs.* lowly expressed genes in *Gryllus bimaculatus*, *Oncopeltus fasciatus*, and *Parhyale hawaiensis*

		*Gryllus*		*Oncopeltus*		*Parhyale*	
GC3/AT3 optimal codons		AT3		AT3		GC3	
No. optimal codons		17		16		13	
Amino acid	Codon	∆RSCU	*P**^a^*	∆RSCU	*P**^a^*	∆RSCU	*P**^a^*
Ala	GCT	**+0.279**	***	**+0.370**	***	+0.048	
Ala	GCC	−0.060		−0.081	*	**+0.028**	**
Ala	GCA	−0.055		−0.120	**	+0.012	
Ala	GCG	−0.123	***	−0.101	**	−0.079	
Arg	CGT	**+0.277**	**	**+0.098**	**	+0.060	
Arg	CGC	+0.013		−0.101	**	−0.001	
Arg	CGA	+0.081		−0.019		−0.051	
Arg	CGG	+0.029		−0.047		−0.163	
Arg	AGA	−0.259	**	+0.050		+0.122	
Arg	AGG	−0.019		+0.117		+0.064	
Asn	AAT	**+0.074**	**	**+0.126**	***	−0.054	**
Asn	AAC	−0.060	*	−0.110	***	**+0.078**	***
Asp	GAT	**+0.132**	***	**+0.129**	***	+0.078	
Asp	GAC	−0.091	**	−0.113	***	−0.031	
Cys	TGT	**+0.170**	**	**+0.216**	***	−0.167	**
Cys	TGC	−0.033		+0.003		−0.013	
Gln	CAA	−0.070	*	+0.063		−0.064	**
Gln	CAG	**+0.097**	**	−0.013		**+0.078**	**
Glu	GAA	**+0.063**	*	**+0.107**	***	−0.005	
Glu	GAG	−0.021		−0.091	**	+0.030	
Gly	GGT	**+0.180**	***	**+0.317**	***	+0.021	
Gly	GGC	−0.018		−0.183	***	+0.017	
Gly	GGA	+0.011		−0.026		**+0.096**	*
Gly	GGG	−0.133	**	−0.074	*	−0.127	***
His	CAT	**+0.129**	**	**+0.102**	**	+0.013	
His	CAC	−0.034		−0.029		−0.035	
Ile	ATT	**+0.311**	***	**+0.167**	***	−0.027	
Ile	ATC	−0.135	**	−0.033		**+0.136**	***
Ile	ATA	−0.125	**	−0.100	**	−0.112	**
Leu	TTA	−0.034		+0.073	*	−0.226	***
Leu	TTG	**+0.300**	***	+0.017		+0.053	
Leu	CTT	+0.068		**+0.346**	***	+0.004	
Leu	CTC	−0.217	***	−0.147	**	**+0.146**	***
Leu	CTA	−0.075		−0.112	**	−0.064	**
Leu	CTG	−0.021		−0.177	***	+0.087	
Lys	AAA	−0.032		+0.019		−0.100	***
Lys	AAG	+0.059		−0.002		**+0.110**	***
Phe	**TTT**	**+0.062***^b^*		**+0.118**	***	−0.075	**
Phe	TTC	−0.015		−0.078	**	**+0.091**	***
Pro	CCT	+0.158*^c^*	*	**+0.194**	**	+0.164	
Pro	CCC	−0.190	***	−0.052		**+0.063**	**
Pro	CCA	**+0.139***^c^*	**	−0.009		−0.108	*
Pro	CCG	−0.038		−0.124	***	−0.129	**
Ser	TCT	**+0.297**	***	**+0.359**	***	+0.015	
Ser	TCC	−0.200	***	−0.142	**	−0.023	
Ser	TCA	−0.024		+0.123	*	+0.038	**
Ser	TCG	−0.007		−0.127	***	**+0.089**	**
Ser	AGT	+0.024		−0.081		−0.079	**
Ser	AGC	−0.090		−0.131	**	−0.040	
Thr	ACT	**+0.163**	**	**+0.244**	***	+0.033	
Thr	ACC	−0.104	**	−0.122	**	**+0.114**	**
Thr	ACA	+0.040		−0.033		−0.091	
Thr	ACG	−0.114	**	−0.080	**	−0.052	
**Tyr**	**TAT**	**+0.081***^b^*		**+0.176**	***	−0.106	
Tyr	TAC	+0.061		−0.140	***	**+0.086***^b^*	
Val	GTT	**+0.243**	***	**+0.261**	***	−0.031	
Val	GTC	−0.141	***	−0.100	**	**+0.081**	*
Val	GTA	−0.126	**	−0.108	*	−0.121	**
Val	GTG	+0.037		−0.041		+0.069	**

The codon identified as the primary optimal codon for each amino acid is in boldface. RSCU, relative synonymous codon usage.

a Asterisks indicate P value using t-tests where **P < 0.05, ***P < 0.001. Codons with *0.05 > *P* < 0.1 are also indicated and considered putative optimal codons. The means and standard errors for highly and for lowly expressed CDS are provided in Table S2. Species are abbreviated using their genus name.

b The codons TTT and TAT for *G. bimaculatus* and TAC for *P. hawaiensis* are identified as candidate optimal codons with *P* values at or slightly above 0.1.

c For the amino acid Pro in *G. bimaculatus*, CCA was selected as the optimal codon due to the fact that it had a lower *P* value than CCT, although both exhibit signals of being optimal codons.

For *P. hawaiensis*, 13 amino acids also were found to have an optimal codon that was favored in highly expressed genes (Table 1). However, in contrast to *G. bimaculatus* and *O. fasciatus*, the majority (12 of 13) of optimal codons were GC3 codons (Table 1). Notably, the absolute value of ∆RSCU was of markedly lower magnitude for *P. hawaiensis* (mean and standard error, 0.0729 ± 0.0062) than for *G. bimaculatus* (0.1060 ± 0.0110) and *O. fasciatus* (0.1158 ± 0.0111) (*P* < 0.016 for *t*-tests). This finding may indicate that selective forces acting on optimal codons are weaker in the amphipod than in the two insects. Nevertheless, collectively the transcript data for all three invertebrate species show evidence of favoritism toward specific synonymous codons in highly expressed genes.

It is worth noting that genes with longer assembled CDS in the aforementioned analyses could have a greater RPM using next-generation sequencing data due to their greater size, and thus not solely result from high expression. Accordingly, as described in File S1, we repeated our analyses using RPKM, which includes length when measuring expression level, and found the same optimal codon lists to that obtained from RPM, with the exception that P-values were weaker. In sum, we show that RPM provides the most rigorous method to identify optimal codons (File S1), and thus use these codon lists for all analyses.

Another complementary method used to further confirm optimal codon lists is to compare codon use between ribosomal protein genes (RPGs), which typically are highly expressed, and lowly expressed genes (Heger and Ponting 2007; Wang *et al.* 2011). Thus, we repeated our analyses of RSCU using RSCU_RPGs_= RSCU_RPGs_ – RSCU_CDS with Lowest 5% Expression_ (File S2) and found that the results of these analyses also support the existence of AT3 optimal codons in *G. bimaculatus* and *O. fasciatus*, and GC3 codons for *P. hawaiensis*.

### Genome-wide optimal codon usage

**Figure 2:**
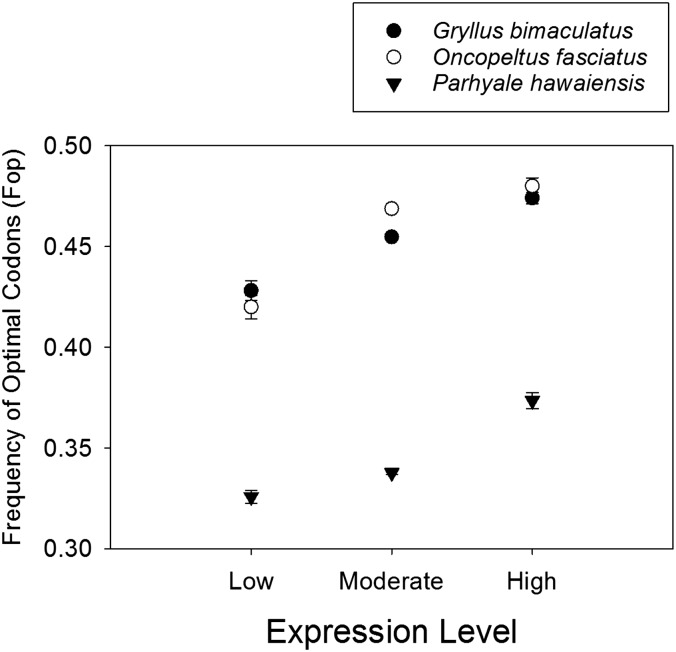
The average frequency of optimal codons (Fop) relative to expression level for the three species of invertebrates. Expression of coding sequence was categorized as low (above 95th percentile), moderate (between 5th and 95th percentile), and high (below 5th percentile). Error bars represent standard errors and are very small.

Using the optimal codon lists in Table 1, we calculated the frequency of optimal codons (Fop) (Ikemura 1981) for each CDS under study and found that this parameter was highly statistically significantly and positively correlated to RPM across all CDS for *G. bimaculatus* (Spearman Rank R = 0.23, *P* < 10^−15^), *O. fasciatus* (R = 0.17, *P* < 10^−15^), and *P. hawaiensis* (R = 0.09, *P* < 10^−15^). The R values were each <0.3, suggesting a moderate, yet highly significant, association between Fop and RPM. We subsequently binned each CDS into one of three distinct RPM categories, namely low (below the 5th percentile), moderate (between the 5th and the 95th percentile), and high (above the 95th percentile). This approach revealed an unambiguous shift in Fop, increasing progressively from the low, moderate to high RPM classes for *G. bimaculatus*, *O. fasciatus*, and *P. hawaiensis* (Figure 2), a trend consistent with a strong connection between transcription rates and Fop. Thus, although selection on codon usage has greatest effects in the uppermost expression levels, it also shapes codon usage (albeit to a lesser extent) in moderately expressed genes in those taxa. The weakest effect appears to be for moderately expressed genes in *P. hawaiensis*, which was only modestly higher than the low expression class. Thus, optimization of codon usage for the moderate expression level classes may be mild in this taxon given the relatively low absolute value of the genome-wide R value above, the similarity of Fop to the lowest class (Figure 2), and the fact that ENC showed an effect only in the highest expression category (see *Identification of optimal codons*). Given these trends, it is evident that the method of contrasting of expression among the CDS with the 5% highest and lowest RPM used herein, was advantageous in revealing the list of optimal codons. This agrees with prior research indicating that comparison of codon usage among the highest and lowest expressed CDS (known as the comparison method) provides a more effective tool (not weakened by mild correlations in the mid-ranges of expression) for revealing optimal codons than broad correlations between codon usage and expression across all CDS (known as the correlation method), at least for these particular organisms (Hershberg and Petrov 2009; Wang *et al.* 2011).

We also report that strong positive correlations were found between Fop and AT3 for *G. bimaculatus* (Spearman R = 0.62, *P* < 10^−15^) and *O. fasciatus* (R = 0.82. *P* < 10^−15^) and between Fop and GC3 (R = 0.64. *P* < 10^−15^) for *P. hawaiensis* (Figure S1). This affirms the strong link between these traits, even in the lattermost taxon where only 12 GC3 optimal codons were identified.

### Optimal codon usage varies among amino acids

The strength of bias in codon usage varied among amino acids. Specifically, the degree of biased codon usage in the 5% most highly and least highly expressed genes was strongest for the highly degenerate amino acids. For *G. bimaculatus*, the three amino acids with six synonymous codons tended to have the largest ∆RSCU among highly and lowly expressed genes: Arg (+0.277), Leu (+0.300), and Ser (+0.297) (Table 1; see Table S2 for means and standard errors per expression category), whereas three- and four-fold degenerate amino acids (Ala, Gly, Ile, Pro, Thr, and Val) ranged between mean ∆RSCU values of +0.163 and +0.311 (Table 1). In contrast, for amino acids with two-fold degeneracy (Asn, Asp, Cys, Gln, Glu, His, Phe, and Tyr) the ∆RSCU values ranged between +0.062 to +0.170 (Table 1). Thus, it appears the selective pressure favoring the use of optimal codons increases with greater degeneracy. Similarly, for *O. fasciatus*, the highest ∆RSCU values were found for the six-fold degenerate amino acids Leu (+0.346) and Ser (+0.359) and the four-fold degenerate amino acid Ala (+0.370), whereas the lowest values included the two-fold amino acids His (+0.102) and Phe (+0.118). Thus, as found for *G. bimaculatus*, these milkweed bug data are consistent with a tendency for greater selective pressure on use of optimal codons for more degenerate amino acids. However, this relationship is not universal in *O. fasciatus*, as ∆RSCU for Arg was +0.098 in this taxon (Table 1). The trends in these two hemimetabolous insects could indicate a hierarchy of selection coefficients on codon usage (Cutter, *et al.* 2006; McVean and Vieira 1999), which is greater in amino acids with higher degeneracy. For the taxon *P. hawaiensis*, the highest ∆RSCU occurred for Leu (+0.146) and Ile (+0.136), but the remaining amino acids showed no trends with respect to degeneracy. This may reflect the fact that optimal codon usage and ∆RSCU were weakest in this taxon, making differences among amino acids accordingly less marked.

### Optimal codon usage is shaped by selection

It has been observed that although codons with elevated frequency in highly expressed genes typically result from selection, they also could result from mutational biases (Comeron 2004). For instance, this might occur if high transcription rates lead to a bias toward specific mutations (*e.g.*, C to T) in the DNA strand, which has been observed in *Escherichia coli* (Beletskii and Bhagwat 1996, 1998) and/or from biases arising during transcriptional-coupled repair such as those reported in mammals (Green *et al.* 2003). Traditionally, one method used to exclude mutational bias is to compare GC content of introns *vs.* GC3 frequency in CDS, where the absence of a relationship excludes mutational bias (Bachtrog 2007; Comeron 2004; Qiu *et al.* 2011; Williford and Demuth 2012). As the RNA-seq data we have studied here does not contain introns, we cannot test for putative mutation bias using this method. Nonetheless, several features of our data point toward selection as a significant factor in the evolution of optimal codons in these taxa, rather than an expression-induced mutational bias. First, for *G. bimaculatus* and *O. fasciatus* the vast majority of codons defined as optimal in Table 1 end in T. However, examining amino acids with four-fold degenerate sites (Ala, Gly, Pro, Thr, Val), which can end in A, G, C, or T, it is evident that there is substantial variation in the ∆RSCU of the optimal codon across amino acids. This finding is not consistent with mutational bias, as all possible types of mutations to T (G to T, C to T, A to T) can occur at the degenerate site. If any neutral mutational bias to T3 were responsible for the evolution of optimal codons for these amino acids, then such bias should affect all five of these amino acids similarly and thus should lead to similar ∆RSCU values. However, these values in fact ranged from +0.139 to +0.279 for *G. bimaculatus*, and from +0.194 to +0.370 for *O. fasciatus* (Table 1). Second, the very large positive ∆RSCU values for four- and six-fold degenerate amino acids (as compared to two-fold) are consistent with selection (McVean and Vieira 2001; Cutter *et al.* 2006) rather than mutational bias. Third, for the six-fold degenerate amino acid Ser, which has two codons ending in T (TCT, AGT), we only observed a strong signal of optimal codons for TCT and not AGT in *G. bimaculatus*; this was also the case for *O. fasciatus*. The presence of a C to T mutational bias would be expected to result in both TCT (from TCC to TCT mutations) and AGT (from AGC to AGT mutations) having a statistically significant and positive ∆RSCU. However, we observed no such effect at the AGT codon (+0.024 for *G. bimaculatus* and −0.081 for *O. fasciatus*, neither of which were significant; Table 1). We note that the caveat for this particular finding for Ser is that it excludes solely a C to T mutational bias in these species. Collectively the aforementioned results are suggestive that factors other than mutational bias, namely selective pressures, contribute toward shaping the optimal codons for *G. bimaculatus* and *O. fasciatus*. Nonetheless, we do not exclude that some mutational pressures, such as neighboring-nucleotide effects, could contribute to nucleotide composition (Hodgkinson and Eyre-Walker 2011). Finally, for *P. hawaiensis*, where the majority of optimal codons ended in GC, the ∆RSCU for the optimal codon identified for amino acids with four synonymous codons varied extensively (+0.028 to +0.114), whereas one ended in A. Both of these observations are inconsistent with mutational bias as an underlying cause of optimal codons. In sum, our data point toward selection as a factor shaping codon usage in all three arthropods.

### Optimal codon usage correlates to amino acid size and complexity

We assessed the cost of protein synthesis using the Dufton (1997) methodology, wherein each amino acid is assigned a size complexity score (S/C) based on its molecular weight and complexity. The S/C score reflects the chemical energy investment, as well as the costs of stability in the protein’s final conformation (Dufton 1997; Williford and Demuth 2012). The S/C scores are listed in Table S3. For each species, we calculated the proportion of amino acids per protein with high S/C (>40; Pr_High SC_), which include Cys, Phe, His, Met, Arg, Trp, and Tyr. We found that Pr_High SC_ was inversely correlated with Fop for *G. bimaculatus* (Spearman R = −0.09, *P* < 10^−15^) and *O. fasciatus* (Spearman R = −0.12, *P* < 10^−15^) (see below in this section for *P. hawaiensis*). Given that codon adaptation indices, such as Fop, correlate with expression and can be used as a proxy to measure expression levels of genes across the genome (Coghlan and Wolfe 2000; Drummond *et al.* 2005; Popescu *et al.* 2006; Wall *et al.* 2005; Williford and Demuth 2012), we can infer that higher gene expression is linked to reduced S/C score in these insects. The R values are similar to those reported for expression and mean S/C in the flour beetle *T. castaneum* (Williford and Demuth 2012) and suggest that selection for reduced S/C is not exclusive to highly expressed genes but rather exhibits a gradual decline with reduced transcription rates. Nonetheless, our data show that the relationship is not particularly strong as revealed by the absolute value of R (≤0.12), possibly indicating that the selection pressure on optimal codon usage relative to S/C varies among genes. In other words, S/C may be under relatively greater selective pressure in some genes than Fop and vice-versa.

**Figure 3:**
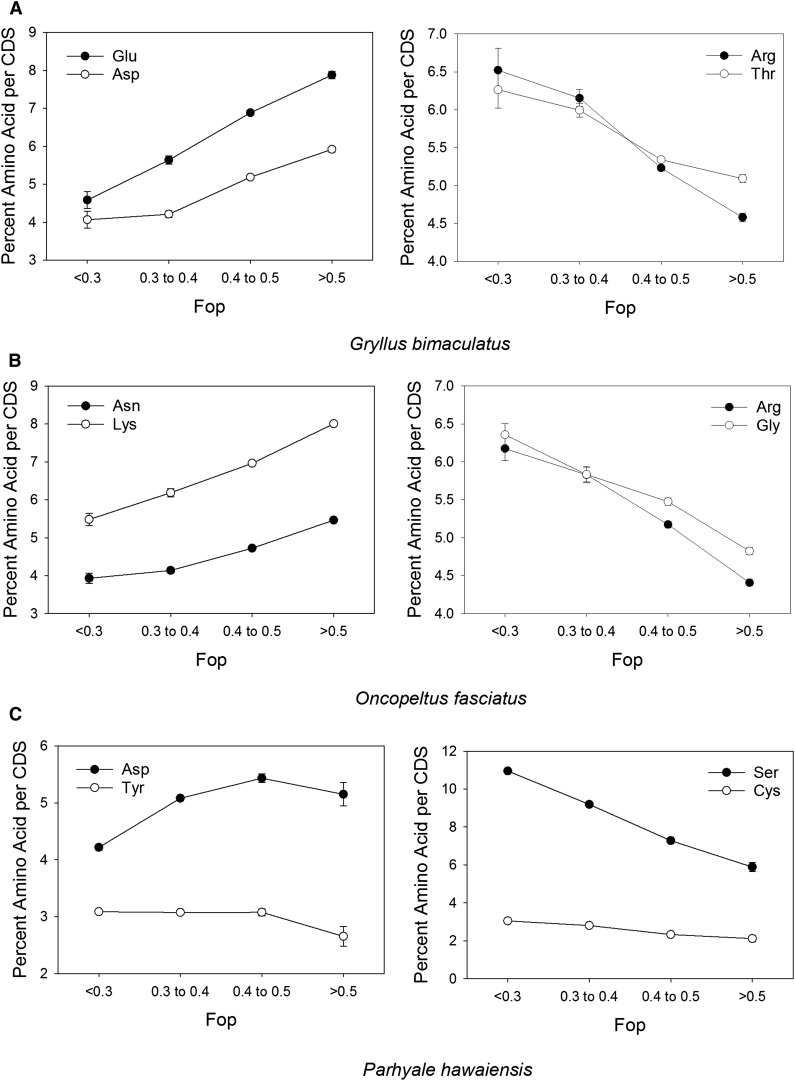
The two amino acids with the largest positive (left) and negative (right) correlation to Fop/expression level in (A) *G. bimaculatus*; (B) *O. fasciatus*; and (C) *P. hawaiensis*. Fop was binned into four categories as shown. Spearman R correlations in Table 2 were calculated with the use of all (unbinned) data points.

We further studied the relationship between expression and the frequency of each of the individual 20 amino acids. For this, we used Fop as a proxy for the relative expression level at the genome-wide level (Coghlan and Wolfe 2000; Drummond *et al.* 2005; Popescu *et al.* 2006; Wall *et al.* 2005), which is apt to be less noisy than RPM or RPKM at intermediate expression levels (outside the upper and lower 5%; see *Identification of optimal codons*). In addition, we wished to assess the relationship between Fop and amino acid in and of itself. The Spearman rank correlations between Fop and amino acid frequency per CDS at the genome-wide level are shown in Table 2. The results showed that for *G. bimaculatus*, 15 amino acids exhibited a statistically significant correlation. The negative R values, which indicate amino acids used rarely in genes with high Fop/expression level, include four of the six amino acids with high S/C scores (>40, Table S3) namely Arg, Met, His, and Trp (the latter two are nonsignificant), consistent both with the inverse relationship described above between Pr_High SC_ and Fop (see *Optimal codon usage correlates to amino acid size and complexity*), and suggest selection against these amino acids in highly expressed genes. The positive correlations indicate that the more frequently an amino acid appeared in a CDS sequence, the more likely it was to display elevated optimal codon usage. Accordingly, the amino acids most favored under high expression (with R > 0.269, *P* < 10^−15^) included Glu and Asp, which have moderate S/C scores (between 32.7 and 36.5, Table S3). The amino acids Asn, Lys and Ile also exhibited substantial positive correlations with frequency in CDS and expression levels (R = 0.169, 0.151, and 0.115, respectively, *P* < 10^−15^), and have moderate or low S/C scores (33.7, 30.1, and 16.0, respectively, Table S3). The two amino acids with the largest positive R values with respect to Fop, namely Glu and Asp, and those with the most negative values, Arg and Thr, are illustrated in Figure 3A (Fop values are binned into four distinct categories: Fop < 0.3, ≥3 Fop < 0.4, ≥0.4 Fop < 0.5, and Fop ≥0.5). These results further confirm the striking shifts in amino acid frequency under high Fop/expression. In summary, it is evident that specific amino acids are preferred under high expression in *G. bimaculatus*, and these tend to be of moderate or low size and complexity.

**Table 2:** The Spearman rank correlations between the frequency of each amino acid per CDS and the Fop

Amino acid*^a^*	*Gryllus*, R	*P*		*Oncopeltus*, R	*P*		*Parhyale*, R	*P*
Arg	−0.198	<0.001	Arg	−0.252	<0.001	Ser	−0.377	<0.001
Thr	−0.123	<0.001	Gly	−0.188	<0.001	Cys	−0.132	<0.001
Pro	−0.122	<0.001	Ala	−0.183	<0.001	Thr	−0.119	<0.001
Ser	−0.110	<0.001	Pro	−0.161	<0.001	Leu	−0.102	<0.001
Ala	−0.093	<0.001	Leu	−0.183	<0.001	Arg	−0.021	0.085
Leu	−0.083	<0.001	Thr	−0.099	<0.001	Pro	−0.021	0.079
Gly	−0.061	<0.001	Met	−0.061	<0.001	Val	−0.011	0.347
Met	−0.049	<0.001	Trp	−0.059	<0.001	Asn	−0.008	0.512
Gln	−0.019	0.155	Val	−0.054	<0.001	His	−0.003	0.778
Trp	−0.008	0.571	Ser	−0.038	0.003	Ile	0.034	0.005
His	−0.003	0.850	Gln	−0.032	0.012	Ala	0.060	<0.001
Cys	0.029	0.033	His	−0.020	0.110	Gln	0.065	<0.001
Val	0.040	0.003	Cys	0.035	0.006	Trp	0.089	<0.001
Phe	0.050	<0.001	Tyr	0.059	<0.001	Phe	0.090	<0.001
Tyr	0.079	<0.001	Phe	0.085	<0.001	Met	0.103	<0.001
Ile	0.115	<0.001	Ile	0.198	<0.001	Glu	0.114	<0.001
Lys	0.151	<0.001	Glu	0.211	<0.001	Lys	0.121	<0.001
Asn	0.169	<0.001	Asp	0.226	<0.001	Gly	0.141	<0.001
Asp	0.269	<0.001	Lys	0.272	<0.001	Tyr	0.158	<0.001
Glu	0.270	<0.001	Asn	0.287	<0.001	Asp	0.195	<0.001

Species are abbreviated using their genus name. CDS, coding sequence; Fop, frequency of optimal codons.

a Amino acids are listed from the most negative to positive R values for each species.

In *O. fasciatus*, 16 amino acids exhibited a statistically significant change in frequency with Fop/expression level (Table 2). The two amino acids with the strongest negative (Arg, Gly) and strongest positive (Asn, Lys) R values are illustrated in Figure 3B. The strongest negative correlation was for Arg (R = −0.252, *P* < 10^−15^), which also has among the largest S/C scores (56.3), implying losses of this metabolically expensive amino acid in CDS expressed at high levels (Table 2, Figure 3B). However, Gly and Ala also exhibited substantial inverse correlations (between −0.188 and −0.183, *P* < 10^−15^) and have low S/C scores (between 1 and 4.8), implying reduced frequency of these amino acids despite their very low metabolic costs. The amino acids exhibiting the strongest positive correlations with Fop/expression levels were Asn, Lys, Asp, and Glu (R between 0.211 and 0.287, *P* < 10^−15^; Table 2, Figure 3B). Remarkably, each of these amino acids has S/C scores within a narrow range from 30.1 to 36.5. Thus, similar to *G. bimaculatus*, genes expressed at high levels appear to favor not amino acids with the lowest S/C scores, but rather a narrow range of moderately sized amino acids, potentially indicating an advantage to maintaining an intermediate protein size under high expression, rather than the smallest possible size. Thus, this explains the negative association between Pr_High SC_ and Fop reported previously (see preceding paragraph in this section), *i.e.*, an average decline in use of amino acids with extreme S/C in highly expressed genes, while indicating this decline does not necessarily favor the lowest S/C amino acids in these invertebrates. Notably, eight of the nine amino acids with positive R in *G. bimaculatus* also had positive R in *O. fasciatus*, implying similar preferences for specific amino acids among these divergent insects from different orders (Orthoptera and Hemiptera, respectively). Furthermore, the four amino acids with strongest positive R values (Lys, Asn, Asp and Glu) were identical among these two arthropods (Table 2), implying a shared preference for intermediate sized amino acids under high expression. In the beetle *T. castaneum* (Coleoptera), it has been reported that amino acids with moderate and with low scores were preferred under high expression (*e.g.*, Glu, Asp, Lys, Val, Ala, and Gly) (Williford and Demuth 2012), whereas our data for *G. bimaculatus* and *O. fasciatus* reveal a strong preference for intermediate sized amino acids (excluding the lowest classes), suggesting species-specific effects on amino acid preferences.

Together, these results from individual amino acids in *G. bimaculatus* and *O. fasciatus* indicate that the moderate negative correlation between Pr_High SC_ and Fop is due to a shift toward increased usage of intermediate cost amino acids, coupled with a decline in both very high and low cost amino acids under high expression. It is worth noting that degeneracy was sometimes lower for higher cost amino acids (*e.g.*, Phe *vs.* Ser, Table S3), which could conceivably contribute to an inverse correlation between Pr_High SC_ and Fop (since codon bias was reduced for low degeneracy amino acids, Table 1). However, our results in Table 2 show that degeneracy effects on Fop did not underlie the Pr_High SC_ and Fop negative correlation. For example, for *G. bimaculatus*, the two-fold degenerate amino acids Phe (S/C score 44.0) and Cys (57.2) exhibited positive (rather than negative) correlations with expression while highly degenerate amino acids such as Ser (S/C score of 17.8) exhibited a large negative correlation (rather than positive) with transcription/Fop level (Table 2). We therefore propose that our findings with respect to Pr_High SC_ cannot result from an artifactual relationship between Fop and amino acid usage. Rather, our data suggest that the reduced usage of expensive amino acids is primarily caused by a greater frequency of moderate cost amino acids under high expression.

For *P. hawaiensis*, the relationship between Fop and Pr_High SC_ was weakly positive (Spearman’s R = 0.050, *P* < 10^−15^), consistent with a mild tendency for higher protein size under high expression, and implying a more complex dynamic between expression and protein size in this taxon. This is supported by the relationships between Fop/expression level and amino acid frequencies per gene. Specifically, Fop/expression level was inversely correlated with the frequency of several amino acids per CDS, including Ser, Cys, Thr, and Leu (R between −0.377 and −0.102, *P* < 10^−15^). In turn, the strongest positive correlations were found for Asp, Tyr, Gly, Lys, Glu and Met (R between 0.103 and 0.195, *P* < 10^−15^). Thus, high Fop/expression level in *P. hawaiensis* genes favors use of a distinct set of amino acids as compared to *G. bimaculatus* and *O. fasciatus*. As shown in Figure 3C, when binned into four distinct classes of Fop values, the amino acids with the most positive (Asp) and negative (Ser) R values exhibited a clear link to Fop/expression level, but those with the second largest value (Tyr and Cys, respectively) exhibited a much weaker effect. Further, some amino acids that were more frequent under high Fop/expression level showed no clear trend in S/C score. For example, Tyr (with positive R) has among the highest S/C scores (57), whereas Gly (also with positive R) exhibits the lowest S/C score (1) (Table 2 and Table S3). This might indicate that a balance of large and small amino acids are preferred under elevated expression *in P. hawaiensis*, that codon and amino acid usage are subject to much weaker selection outside the highest expressed gene set (see *Genome-wide optimal codon usage*), or that factors other than protein size play a predominant role in shaping amino acid frequency in this organism.

### Codon usage and CDS lengths in *D. melanogaster* orthologs support translational selection

**Figure 4:**
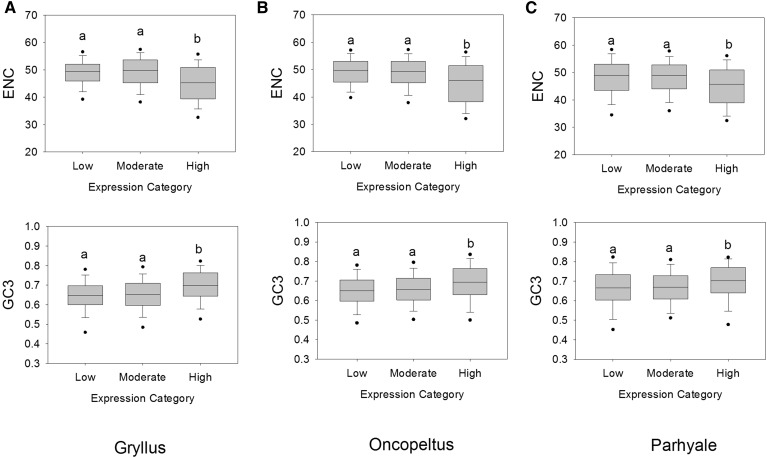
Bar and whisker plots of the effective number of codons (ENCs) and GC3 content of *D. melanogaste*r orthologs to coding sequence with low, moderate, and high expression in (A) *G. bimaculatus*; (B) *O. fasciatus*; and (C) *P. hawaiensis*. *P*-values of ranked analysis of variance <0.0003 for each figure. Different letters in each figure indicate paired differences using Dunn’s contrast (*P* < 0.05).

We evaluated the role of CDS length and GO annotation category in our findings using the well-studied and annotated model system *D. melanogaster* (Graveley *et al.* 2011; St Pierre *et al.* 2014) as a reference system. First, we determined whether genes exhibiting optimal codon usage in each of our three arthropods under study also exhibit optimal codon usage in their orthologs in *D. melanogaster*, a species previously shown to have GC3 optimal codons (Duret and Mouchiroud 1999). Thus, GC3 provides an effective measure of optimal codon usage in this taxon. Note that we did not cross compare the CDS sets of *G. bimaculatus*, *O. fasciatus* and *P. hawaiensis* since, given the *de novo* assembled transcriptomes that we used, apparent losses of orthologs among each pair might result from any one of expression below detection thresholds, assembly methods, or true gene loss, thereby minimizing gene sets; this ambiguity would not occur in *D. melanogaster*, which has a complete annotated gene list. Thus, we independently contrasted each gene set to their orthologs in *D. melanogaster*. The goal of this analysis was to determine whether orthologs to highly expressed genes in each of the three species under study, also exhibited elevated optimal codon usage in a divergent taxon. The number of *D. melanogaster* orthologs matching the CDS list from *G. bimaculatus*, *O. fasciatus* and *P. hawaiensis* was 3960 (74.9% of the CDS set under study), 4190 (68.0%) and 2822 (41.9%), respectively. Using the *D. melanogaster* orthologs of those CDS from the low (lowest 5% RPM), moderate (>5% RPM < 95%), and high (highest 5% RPM) categories in each of our three study species, we measured the ENC. We then evaluated the ENCs and GC3 (optimal codon) values across the three expression level categories. As shown in Figure 4A, the *D. melanogaster* orthologs of the *G. bimaculatus* genes in the highest expression category showed markedly lower ENC and higher GC3 than those from the low or moderate categories (*P*-value ranked analysis of variance [ANOVA] were 2.2 × 10^−15^ and 2.2 × 10^−15^, respectively, Dunn’s *post hoc* tests *P* < 0.05). This confirms optimization of codon usage in the highly expressed gene set across these divergent organisms, bias toward AT3 codons in *G. bimaculatus* and GC3 in *D. melanogaster* (note: higher expression in *D. melanogaster* is evident based on elevated optional codon usage (defined here as GC3), (Coghlan and Wolfe 2000; Drummond *et al.* 2005; Popescu *et al.* 2006; Wall *et al.* 2005; Williford and Demuth 2012). For *O. fasciatus*, identical trends were detected (Figure 4B), wherein *D. melanogaster* orthologs of the high expression dataset had lower ENC and higher GC3 than those from the low and moderate classes (P-ranked ANOVA = 4.2 × 10^−11^ and 6.9 × 10^−7^, respectively, Dunn’s *post hoc* tests *P* < 0.05). Finally, for *P. hawaiensis*, lower ENC and higher GC3 was also observed for orthologs matching the high expression category genes, than for the low and moderate expression categories (*P* = 5.8 × 10^−7^ and 3.3 × 10^−4^, respectively; Dunn’s *post hoc* tests *P* < 0.05) (Figure 4C), indicating a GC3 bias in each of these arthropods. Collectively, these results demonstrate that enhanced optimal codon usage in the high expression CDS dataset of *G. bimaculatus*, *O. fasciatus* and of *P. hawaiensis* is shared with their orthologs from the divergent relative *D. melanogaster*.

We next evaluated the relationship between CDS length and gene expression level for each arthropod under study, using lengths obtained from *D. melanogaster* orthologs. This is an effective approach as protein lengths tend to be highly conserved in eukaryotes (Wang *et al.* 2005), and was used since transcriptome assembly may not always yield full CDS lengths. Our objective was to determine whether high expression is linked to shorter/longer protein lengths. As shown in Figure S2, the length (number of codons) of the *D. melanogaster* orthologs matching CDS from *G. bimaculatus*, *O. fasciatus* and in *P. hawaiensis* were shortest for the high expression category, intermediate for moderate class, and longest for the lowest expression category for each of the species under study (ranked ANOVA *P* < 2.9 × 10^−9^ for all three species). This pattern indicates that translational selection favors short proteins as a function of expression (Figure 4). Taken together, our collective findings indicate that highly expressed genes encode short proteins (Figure 4), exhibit high Fop (Table 1 and Figure 2), and preferentially use specific amino acids (Table 2), each of which promote cost-efficient translation.

Given that highly expressed genes tend to be shorter than and lower expressed genes (Figure S2), we considered the role of genetic-linkage in our study. Selective sweeps or background selection can lead to fixation of linked nonoptimal codons, which may have greater effects in longer genes, and could reduce Fop (Comeron *et al.* 1999; Loewe and Charlesworth 2007; Whittle *et al.* 2011b). We thus compared Fop of long genes (>500 codons) from the high expression (highest 5% expression) *vs.* those from the low expression (lowest 5%) category for each species under study. We found that, consistent with the results of our analysis for all highly expressed genes regardless of length (Table 1 and Figure 2), Fop was statistically significantly elevated in the high- compared with the low-expression category for long genes in *G. bimaculatus* (Mean_High_ = 0.483 ± 0.003, Mean_Low_= 0.441 ± 0.007 MWU *P* < 10^−15^), *O. fasciatus* (Mean_High_ = 0.497 ± 0.005, Mean_Low_= 0.426 ± 0.008 MWU *P* < 10^−15^), and in *P. hawaiensis* (Mean_High_ = 0.409 ± 0.013, Mean_Low_= 0.348 ± 0.009 MWU *P* = 0.0002). This argues against a pervasive role of genetic linkage, which, were it the major factor shaping codon use evolution, would be expected to yield similar Fop in long genes from both the high and low expression categories. Thus, long genes appear most influenced by their expression level, and are under greater selective pressure on codon usage when highly transcribed. Accordingly, we propose that genetic linkage, if it has an effect on Fop in these organisms, is largely restricted to the lower expression classes, where CDS are on average markedly longer (Figure S2) and purifying selective pressures are apt to be weaker due to their low transcription (Subramanian and Kumar 2004).

### Functional ontology annotation shows highly transcribed CDS are involved in cell-cycling and translation

We wished to determine whether highly expressed genes with elevated optimal codon usage were preferentially associated with particular functions, biological processes or cellular components. To this end, we used *D. melanogaster* orthologs to assess GO annotation (Ashburner *et al.* 2000) of the CDS in all three arthropods under study. We clustered genes from the highest expression category (above the 95th percentile) in each species into functional groups using the procedures in DAVID (Huang da *et al.* 2009a,b). The results in Table 3 show that high expression was associated with enrichment of genes involved in cell-cycle processes (*e.g.*, spindle organization, microtubule organization, proteosomes), translation (ribosome synthesis, RPGs) and DNA/RNA binding in all three arthropods under study, consistent with roles in rapid cell divisions in the embryo and/or reproductive tissues. Thus, the optimization of codon and amino acid usage observed in these organisms could be instrumental in minimizing the protein biosynthetic costs of cell division and protein turnover in the embryo and sexual tissues (Table S1). Remarkably, translational efficiency includes optimization of codons and amino acids not only in genes involved in cell division, but also in genes controlling protein synthesis itself (RPGs). This latter finding is concordant with extensive literature showing that protein synthesis genes are typically among the highest expressed in an organism (see for example Sharp and Li 1987; Wang *et al.* 2011).

**Table 3:** Functional clustering of the most highly expressed CDS (above the 95th percentile) for each of three arthropod species under study using their orthologs in *Drosophila melanogaster* and the gene ontology system DAVID (Huang da *et al.* 2009a,b)

*Gryllus bimaculatus*	*P* Value*^a^*	*Oncopeltus fasciatus*	*P* Value	*Parhyale hawaiensis*	*P* Value
**Enrichment Score*^b^*: 3.91**		Enrichment Score: 13.46		Enrichment Score: 10.7	
Proteasome regulatory particle	6.20E^–5^	Mitotic spindle organization	5.70E^–18^	Ribonucleoprotein	3.20E^–16^
Proteasome accessory complex	8.00E^–5^	Mitotic cell cycle	2.70E^–17^	Ribosomal protein	6.80E^–16^
Proteasome complex	3.70E^–4^	Spindle organization	3.80E^–17^	Structural constituent of ribosome	3.00E^–13^
**Enrichment Score: 3.9**		Microtubule cytoskeleton organization	2.40E^–16^	Ribosomal subunit	7.90E^–11^
Spindle organization	6.30E^–6^	Microtubule-based process	1.20E^–14^	Ribosome	1.10E^–10^
Cell-cycle process	3.80E^–5^	Cell cycle	2.10E^–12^	Cytosolic ribosome	3.80E^–10^
Mitotic spindle organization	7.10E^–5^	M phase	8.60E^–12^	Ribosome	6.10E^–10^
Mitotic cell cycle	8.10E^–5^	Cytoskeleton organization	1.90E^–11^	Structural molecule activity	1.20E^–9^
Cell cycle	1.20E^–4^	Cell-cycle process	2.00E^–11^	Large ribosomal subunit	1.70E^–9^
Microtubule cytoskeleton organization	2.20E^–4^	Cell-cycle phase	2.50E^–11^	Cytosolic part	3.70E^–8^
Microtubule-based process	4.40E^–4^	Enrichment Score: 6.03		Enrichment Score: 7.13	
M phase	5.70E^–4^	Cytosolic ribosome	7.40E^–9^	Mitotic spindle elongation	5.40E^–12^
Cell-cycle phase	9.30E^–4^	Ribonucleoprotein	9.20E^–9^	Spindle elongation	6.50E^–12^
**Enrichment Score: 3.64**		Ribosomal protein	1.30E^–8^	Mitotic spindle organization	4.10E^–10^
Proteasome	1.00E^–5^	Ribosome	4.10E^–8^	Microtubule cytoskeleton organization	2.00E^–9^
Proteasome complex	3.70E^–4^	Cytosolic large ribosomal subunit	9.50E^–8^	Spindle organization	4.50E^–9^
Proteasome	3.20E^–3^	Structural constituent of ribosome	1.80E^–6^	Microtubule-based process	9.90E^–8^
**Enrichment Score: 3.44**		Ribosomal subunit	1.60E^–5^	Mitotic cell cycle	2.50E^–7^
atp-binding	1.20E^–6^	Large ribosomal subunit	5.10E^–5^	Cytoskeleton organization	2.60E^–7^
Nucleotide-binding	5.70E^–6^	Ribosome	6.90E^–5^	M phase	2.20E^–5^
Adenyl nucleotide binding	8.80E^–4^	Structural molecule activity	1.40E^–3^	Cell-cycle phase	3.70E^–5^
Purine nucleoside binding	9.70E^–4^	Enrichment Score: 4.44		Cell-cycle process	1.40E^–4^
ATP binding	9.80E^–4^	Nucleotide-binding	5.40E^–8^	Cell cycle	2.50E^–4^
Adenyl ribonucleotide binding	1.00E^–3^	Atp-binding	1.70E^–7^	Enrichment Score: 3.63	
Nucleoside binding	1.10E^–3^	Nucleotide binding	7.40E^–6^	Transit peptide	4.30E^–5^
Purine nucleotide binding	1.70E^–3^	Purine nucleotide binding	5.90E^–5^	Mitochondrion	4.50E^–5^
Ribonucleotide binding	1.90E^–3^	Purine ribonucleotide binding	7.50E^–5^	Transit peptide:mitochondrion	6.60E^–3^
Purine ribonucleotide binding	1.90E^–3^	Ribonucleotide binding	7.50E^–5^	Enrichment Score: 3.26	
**Enrichment Score: 3.31**		ATP binding	2.40E^–4^	Proteasome	1.30E^–4^
PINT	1.20E^–4^	Adenyl ribonucleotide binding	2.50E^–4^	Proteasome	9.60E^–4^
Proteasome component region PCI	1.90E^–4^	Adenyl nucleotide binding	4.30E^–4^	Proteasome complex	1.30E^–3^
Domain:PCI	5.40E^–3^	Purine nucleoside binding	4.80E^–4^	Enrichment Score: 2.59	
**Enrichment Score: 2.96**		Nucleoside binding	5.50E^–4^	PINT	4.90E^–4^
Chaperone	5.4E^–4^	Enrichment Score: 4.13		Proteasome component region PCI	8.80E^–4^
Chaperonin TCP-1	7.4E^–4^	Chaperone	8.0E^–4^	Domain:PCI	4.00E^–2^
Chaperonin-containing T-complex	1.40E^–3^	Chaperonin TCP-1	1.4E^–4^		
Chaperonin Cpn60/TCP-1	2.50E^–3^	Chaperonin-containing T-complex	2.70E^–5^		
**Enrichment Score: 2.74**		Chaperonin Cpn60/TCP-1	7.60E^–5^		
Ribosomal protein	9.80E^–5^	PIRSF002584:molecular chaperone t-complex-type	4.00E^–4^		
Ribonucleoprotein	1.00E^–4^	Chaperone	1.70E^–3^		
Cytosolic large ribosomal subunit	2.20E^–4^	Enrichment Score: 3.91			
Cytosolic ribosome	4.30E^–4^	Proteasome	3.60E^–6^		
Structural constituent of ribosome	2.00E^–3^	Proteasome complex	7.00E^–4^		
Ribosome	2.50E^–3^	Proteasome	7.60E^–4^		
Large ribosomal subunit	3.70E^–3^	Enrichment Score: 2.54			
Ribosomal subunit	1.10E^–2^	Proteasome regulatory particle	2.40E^–3^		
Ribosome	2.60E^–2^	Proteasome regulatory particle	3.00E^–3^		
Structural molecule activity	8.20E^–2^	Proteasome accessory complex	3.50E^–3^		

CDS, coding sequence.

a *P*-values represent a modified Fisher’s test, wherein lower values indicate greater enrichment.

b Functional categories with enrichment values >2.5 are shown.

For the CDS pooled from the moderate and low expression categories, we found enrichment of certain DNA/RNA and protein synthesis genes, primarily from the upper expression levels in the moderate class. However, the CDS with moderate and low expression levels spanned a vast range of functionalities rather than showing significant enrichment for any specific GO category compared with the high expression category (Table S4). Such a distribution of GO categories is expected for highly complex eukaryotic embryo/sexual tissues, which are typically comprised of an array of distinct cell types and transcripts (Combs and Eisen 2013; Diez-Roux *et al.* 2011; Donoughe and Extavour 2015; Ewen-Campen *et al.* 2011; Gilboa 2015; Jankovics *et al.* 2014). Although the moderate/low expressed genes are clearly biologically relevant, our data indicate they are less apt to influence codon or amino acid optimization (Figure 2, Figure 3, and Table 2) and have longer CDS lengths (Figure S2), likely due to their reduced transcriptional and translational levels, and thus biosynthetic costs. Together, the consistency in functionalities of the highly expressed dataset in all three arthropods studied herein reveals that optimization of codon and amino acid usage is a shared feature of highly expressed genes, particularly those involved in cell division and translation, in these divergent arthropods.

## Discussion

### Optimal codon and amino acid usage

Major codon and amino acid preferences under high expression point toward adaptation for translational accuracy and/or efficiency (Akashi 2001; Akashi 2003). Here, we identified clear preferences in codon usage and in amino acid frequency relative to expression in three emerging models of arthropods, *G. bimaculatus*, *O. fasciatus*, and *P. hawaiensis*. With respect to optimal codons, preferences have been previously reported in bacteria, fungi such as yeast and Neurospora, and some animals including *D. melanogaster* and *C. elegans*, but remain rare in arthropods outside of the higher insects (Akashi 2003; Duret and Mouchiroud 1999; Whittle *et al.* 2011a). Our results revealed AT3 optimal codons across the vast majority of amino acids in *G. bimaculatus* and *O. fasciatus*, whereas weaker yet significant GC3 preferences were observed in *P. hawaiensis*. Optimal codons have been linked to rapid and accurate translation, the efficient use of ribosomes and a reduced cost of proofreading (Akashi 2001, 2003) and may also improve transcription (Trotta 2011). Thus, the stronger bias in codon usage in highly expressed genes reported here (Table 1) likely reflects the fitness effects of a higher number of transcription and translation events, and in this regard is similar to other invertebrates including *D. melanogaster*, *C. elegans*, and *T. castaneum* (Akashi 2001, 2003; Duret 2000; Williford and Demuth 2012). Given that selection coefficients among synonymous codons are believed to be small, optimal codons may not be as prevalent in organisms with smaller population sizes (Comeron 2004). Thus, our results are consistent with sufficiently large population sizes in each of these three arthropods such that Nes > 1, allowing optimization of codon usage in each organism (Comeron 2004; Cutter *et al.* 2006; Duret and Mouchiroud 1999).

Both mutation and selection can contribute to codon usage. For example, in some organisms such as humans, GC3 content is correlated to GC of adjacent noncoding DNA, suggesting mutational pressures, in addition to selection, play a significant role in its codon usage profiles (Clay *et al.* 1996; Comeron 2004). It is also conceivable that AT3 or GC3 biases correlate to expression because highly transcribed genes are localized to regions that are inherently AT- or GC-rich, respectively. Our present results revealed substantial variation in codon bias among amino acids with the same degeneracy, which combined with the fact that amino acids are likely arbitrarily distributed throughout the genome, supports a role of selection herein. Further, the finding that higher degeneracy is connected to greater biases in codon usage is also consistent with selection, and might indicate that the presence of multiple tRNAs leads to greater pressure for optimization. Nonetheless, future sequence data for introns or other noncoding DNA from all three species under study will help further elucidate the precise role of selection relative to mutation in shaping codon usage in these models of the Pancrustacea.

Selective pressure on amino acids for efficient protein synthesis is much less well studied than codon usage. Revealing preferences for specific amino acids in a range of organism could play a major role in advancing our understanding of the dynamics underlying protein evolution, and thus warrants greater attention. Although some amino acid changes might individually have minor effects on cell physiology or fitness, collectively these effects could explain proteome-wide patterns of amino acid composition and evolution (Akashi 2003). Indeed, amino acid size and complexity has been linked to expression in the invertebrates *D. melanogaster*, *C. elegans* and *T. castaneum* as well as yeast (Akashi 2003; Cutter *et al.* 2006; Williford and Demuth 2012). Our findings extend these conclusions to include *G. bimaculatus* and *O. fasciatus*. However, our results differ from those of other eukaryotes in that we report that highly expressed genes exhibit a prevalence of amino acids with moderate size and complexity, which does not include the smallest classes, suggesting a benefit to utilizing mid-range amino acids, rather than using the smallest in these insects. It is feasible that these amino acids with moderate size/complexity, while more metabolically costly than the smallest, confer an advantage to protein stability or activity.

For *P. hawaiensis*, the dynamic between Fop/expression level and amino acid biosynthesis costs is less straightforward, as amino acids with both high and low S/C scores exhibited statistically significant positive correlations to Fop (Table 2 and Figure 3). This suggests that there is a selective advantage of a mixture of large and small amino acids on protein stability or function in this taxon. However, another interpretation is that only a few amino acids are optimized in this taxon. For instance, the strong positive correlation between Fop/expression and frequency of Asp, and the strong negative correlation for Ser (Table 2 and Figure 3), each demonstrate unambiguous signals of amino acid optimization. However, the weaker signals from the other amino acids (Figure 3) might indicate that the main or strongest effects of expression are limited to Asp and Ser. In addition, the weaker signal of optimal codon usage in *P. hawaiensis*, which is very mild outside the highest expression class, suggests that a more complex dynamic underlies this phenomenon for this taxon. Further studies will be needed to ascertain the dynamics of codon and amino acid usage in this crustacean. Nevertheless, taken together, our data provides evidence of optimization of codon and amino acid usage in highly transcribed genes across all three of these distinct arthropods.

The fact that *D. melanogaster* orthologs to CDS from the high expression category of *G. bimaculatus* and of *O. fasciatus* each had higher GC3 frequencies than orthologs from lower expression classes (Figure 4, A and B), confirms that optimal codon usage is retained across the same gene sets among divergent insects. Thus, despite the fact these various insects have evolved distinct sets of optimal codons, selection for a subset of codons that promote translational efficiency of highly expressed CDS appears to be a conserved trait. Further, our findings showing elevated usage of GC3 optimal codons in highly transcribed CDS of *P. hawaiensis* and their orthologs in *D. melanogaster* (Figure 2 and Figure 4) reveals similar evolution traits across the Pancrustacea. Functional categorization (GO) analyses indicate that for all three species studied herein, the CDS from the highest Fop/expression category primarily included genes involved in cell-cycling and protein synthesis, such as RPGs (which are typically highly expressed) (Wang *et al.* 2011) (Table 3), suggesting that such fundamental processes are especially prone to selection to reduce biosynthetic costs (Seligmann 2003; Swire 2007; Williford and Demuth 2012). Given that transcripts of cell-cycling and translation genes are known to be prevalent throughout embryogenesis and in later developmental phases in arthropods such as *D. melanogaster* and other invertebrates including *C. elegans* (Li *et al.* 2014), optimization of codons and amino acids in these gene sets could potentially markedly reduce lifetime biosynthetic costs (Duret 2000; Seligmann 2003; Swire 2007; Williford and Demuth 2012) and enhance fitness in these organisms.

Our finding that highly expressed CDS encoded short proteins in *G. bimaculatus*, *O. fasciatus*, and in *P. hawaiensis* (Figure S2) concurs with previous studies in eukaryotes such as *D. melanogaster*, *T. castaneum*, humans, yeast, and some plants (Akashi 2001, 2003; Comeron 2004; Ingvarsson 2007; Lemos *et al.* 2005; Moriyama and Powell 1998; Urrutia and Hurst 2001; Whittle *et al.* 2007; Williford and Demuth 2012). The phenomenon of shorter CDS in highly expressed genes might reflect the action of selection to minimize the protein length, which combined with high optimal codon usage and preferential amino acid usage observed herein (Figure 2 and Table 2), potentially reduces the costs of transcription, translation, and cellular transport, in these arthropods (Akashi 2001, 2003). With respect to translational costs, reductions in protein length of highly transcribed genes should have similar advantages to the usage of optimal codons and of reduced-cost amino acids in terms of biosynthesis, suggesting potential for co-evolution of these traits (Akashi 2001, 2003).

The fact that long CDS under high transcription had elevated Fop compared with their lowly transcribed counterparts supports the hypothesis that expression level, rather than genetic-linkage, is the primary factor shaping Fop differences among expression classes. Nevertheless, it is formally possible that genetic linkage may contribute toward the observed reduced Fop in the lower expression level datasets, as longer genes appear most prone to fixation of non-optimal codons (Betancourt and Presgraves 2002; Comeron *et al.* 1999; Loewe and Charlesworth 2007; Whittle and Johannesson 2013). This linkage phenomenon might be most common in genes with weak expression levels, which would be expected to experience lower purifying selection pressures (Subramanian and Kumar 2004). Future data on amino acid substitutions and linked fixations of nonoptimal codons with respect to CDS length among related species in each taxonomic group (*Gryllus*, *Oncopeltus*, and *Parhyale*) will help further discern the role of genetic-interference on Fop (Betancourt and Presgraves 2002; Kim 2004; Whittle and Johannesson 2013). In addition, when available in the future, genomic data for all three of these arthropods will allow precise measures of CDS lengths in all genes under study (not only those with orthologs in *D. melanogaster*), providing a means to test fine-scale gene-length effects on codon usage within expression classes, which to date have typically shown higher codon bias in shorter genes in eukaryotes (Akashi 2001, 2003; Cutter 2008; Duret and Mouchiroud 1999; Moriyama and Powell 1998; Whittle *et al.* 2007, 2011a).

Together, it is evident that at a genome-wide level, high transcription is accompanied by a marked shift toward short protein lengths (Figure S2) and this shift co-occurs with increased usage of optimal codons (Figure 2) and specific amino acids (Table 2 and Figure 3). This points toward multifaceted aspects of translational selection, which affect several genomic traits (codons, amino acids, and CDS length) to yield efficient protein synthesis in these arthropods.

### Fop as a predictor of expression

While Fop, or other codon adaptation indices, have been shown to reliably reflect the expression level in an organism (Coghlan and Wolfe 2000; Drummond *et al.* 2005; Popescu *et al.* 2006; Wall *et al.* 2005), it is worthwhile to note that selection at the protein level can influence optimal codon usage. For instance, at the interspecies level, proteins that evolve rapidly (displaying high nonsynonymous to synonymous substitution rates, dN/dS) (Comeron *et al.* 1999; Loewe and Charlesworth 2007; Whittle and Johannesson 2013; Yang 2007) often exhibit reductions in optimal codon usage. Adaptive evolution at the protein level may cause selective sweeps that fix linked non-optimal codon mutations, and relaxed selection on proteins often correlates to losses in optimal codons (Betancourt and Presgraves 2002; Kim 2004; Sella *et al.* 2009; Whittle *et al.* 2011b). Relaxed selection on proteins or synonymous codons may arise from factors such as reduced population size or lowered recombination rates over an organism’s evolutionary history (Betancourt and Presgraves 2002; Comeron and Kreitman 1998; Haddrill *et al.* 2011; Sella *et al.* 2009; Whittle *et al.* 2011b). However, the relationship between Fop and expression should not be uncoupled by these processes, but rather is likely maintained due to the fact that rates of protein evolution appear to be inversely correlated to expression level in various organisms including mice, fruit fly, and yeast (see for example Drummond *et al.* 2005; Lemos *et al.* 2005; Pal *et al.* 2001; Subramanian and Kumar 2004). The conservation of highly expressed genes at the protein sequence level may be explained by their essentiality, or indispensability, to fitness (Mank and Ellegren 2009). In addition, highly expressed genes might also exhibit signatures of selection on (otherwise) neutral amino acids if such residues are needed to promote translational efficiency, for example because they promote a small size or specific conformation (Akashi 2003). In this regard, Fop and expression level likely remain connected even for genes with rapid rates of protein evolution, such that Fop would decrease under rapid protein evolution, which is more apt to occur in genes with lowered expression. Given that we observed clear relationships between Fop and RPM, and between Fop and the S/C score and amino acid composition (Table 2 and Figure 3), we suggest that in these arthropods, optimal codon usage reflects expression level on a genome-wide level, and that optimal codon use has evolved to promote efficient translation. In this regard, the relationships observed between Fop and amino acid composition would directly reflect adaptation for fast and accurate translation in genes with high expression.

We conclude that highly expressed genes, in particular those involved in cell-cycling and protein synthesis (Table 3), exhibit a history of coevolution of optimal codons (Table 1 and Figure 2), the usage of specific amino acids (Table 2 and Figure 3) and shorter CDS length (Figure S2), which act in concert to promote accurate and cost efficient translation in these divergent arthropods. Our findings indicate that selection favors specific optimal codons and amino acids in three emerging models of arthropods, *G. bimaculatus*, *O. fasciatus*, and *P. hawaiensis*. For both insects, a particularly strong signal of optimal codons was identified. Further, the marked connection between Fop and amino acid size and complexity suggests an adaptive strategy to promote efficient translation, which likely allows fast rates of accurate protein synthesis. Although weaker selective effects on codon usage, with fewer optimal codons, were evident in *P. hawaiensis* compared with the two insects, this taxon also exhibits a significant connection among Fop and size/complexity, suggesting coevolution that appears to favor both small and large amino acids has also occurred in this crustacean. Thus, the dynamics of protein evolution may be more complex in this taxon, wherein protein stability or function in highly expressed genes may necessitate a wide range of amino acids. Further studies should explore the variation in amino acid and synonymous codon mutations and specific types of amino acid replacements at the population level (Akashi and Schaeffer 1997) to reveal whether selective variation can be observed at the intraspecies level in these arthropods. Furthermore, future availability of genomes from these three species will allow further study of other facets of translational selection, including the relationship between tRNA gene abundance and expression, which is particularly relevant to understanding the evolution of optimal codons and its contribution to functional genome evolution.

## 

## Supplementary Material

Supporting Information
