# Supplementary material for: Codon and Amino Acid Usage Are Shaped by Selection Across Divergent Model Organisms of the Pancrustacea
Source: G3 (Bethesda). 2015 Sep 17;5(11):2307–21. doi: 10.1534/g3.115.021402 (PMC4632051; doi:10.1534/g3.115.021402)
Supplement: Supporting Information [file supp_5_11_2307__index.html]

Codon and Amino Acid Usage Are Shaped by Selection Across Divergent Model Organisms of the Pancrustacea — Supporting Information 

# Codon and Amino Acid Usage Are Shaped by Selection Across Divergent Model Organisms of the Pancrustacea

## Supporting Information for Whittle and Extavour, 2015

**Files in this Data Supplement:**

- Supporting Information - Tables S1-S4, Figures S1-S2, Files S1-S3, and Supporting Information References (PDF, 861 KB)
- Table S1 - Transcript datasets used in the present study. (PDF, 107 KB)
- Table S2 - The mean RSCU and standard errors (SE) for highly and lowly expressed genes in *G. bimaculatus*, *O. fasciatus* and *P. hawaiensis*. (PDF, 94 KB)
- Table S3 - The size complexity (S/C) scores per amino acid as per Dufton et al. (1997). (PDF, 87 KB)
- Table S4 - Functional clustering of the pooled moderate and low expressed CDS (all CDS below the 95th percentile of RPM) for each of three arthropod species under study using their orthologs in the model *D. melanogaster*. (PDF, 280 KB)
- Figure S1 - The Spearman rank correlation A) AT3 and Fop for *G. bimaculatus*. B) AT3 and Fop for *O. fasciatus*. C) GC3 and Fop for *P. hawaiensis*. (PDF, 276 KB)
- Figure S2 - Bar and whisker plots of CDS length (number of codons) of *D. melanogaster* orthologs to CDS with low, moderate and high expression in A) *G. bimaculatus*; B) *O. fasciatus*; and C) *P. hawaiensis*. (PDF, 91 KB)
- File S1 - Supporting Results File 1 (PDF, 103 KB)
- File S2 - Supporting Results File 2 (PDF, 103 KB)
- File S3 - Supporting Materials and Methods (PDF, 119 KB)
