# Supplementary material for: Codon and Amino Acid Usage Are Shaped by Selection Across Divergent Model Organisms of the Pancrustacea
Source: G3 (Bethesda). 2015 Sep 17;5(11):2307–21. doi: 10.1534/g3.115.021402 (PMC4632051; doi:10.1534/g3.115.021402)
Supplement: Supporting Information [file supp_g3.115.021402_021402SI.pdf]

G3·Genes | Genomes | Genetics

Codon and amino acid usage are shaped by selection across divergent model organisms of the Pancrustacea

Authors: Carrie A. Whittle[*], Cassandra G. Extavour[*,†]

[*]Department of Organismic and Evolutionary Biology, Harvard University, 16 Divinity Avenue, Cambridge MA 02138, USA

[†]Department of Molecular and Cellular Biology, Harvard University, 16 Divinity Avenue, Cambridge MA 02138, USA

Corresponding author: Cassandra Extavour, Department of Molecular and Cellular Biology, Harvard University, 16 Divinity Avenue, Cambridge MA 02138, USA Email: extavour@oeb.harvard.edu

Identification Numbers of RNA-Seq Data at NCBI: SRR060816, SRR057570, SRR060813

DOI: 10.1534/g3.115.021402

Table S1 Transcript datasets used in the present study. All data per species were used for assembly as described in ASGARD (Ewen-Campen et al. 2011; Zeng et al. 2011; Zeng and Extavour 2012; Zeng et al. 2013). Datasets in bold were used for expression analysis.

	Tissue Type	Sequencing Mode	Sample ID (No. in cited Reference/No. in NCBI)	Library Normalized	No. Reads
G. bimaculatus	Embryos	GS-FLX	SRX023830/SRR060814	Yes	78,936
	Ovaries	GS-FLX	SRX023831/SRR060815	Yes	67,353
	**Pooled Ovaries and Embryos**	**GS_FLX Titanium**	SRX023832/SRR060816	No	4,102,057
				Total	4,248,346
O. fasciatus	**Pooled Ovaries and Embryos**	**GS_FLX Titanium**	SRX022014/ SRR057573	No	1,293,320
	Pooled Ovaries and Embryos	GS_FLX Titanium	SRX022013/ SRR057572	Yes	656, 783
	Embryos	GS-FLX	SRX022012/ SRR057571	Yes	71,912
	Ovaries	GS-FLX	SRX022011/ SRR057570	Yes	65,395
				Total	2,087,410
P. hawaiensis	**Pooled Ovaries and Embryos**	**GS_FLX Titanium**	SRX0238929/SRR060813	No	3,172,925

Table S2 The mean RSCU and standard errors (SE) for highly and lowly expressed genes in *G. bimaculatus*, *O. fasciatus* and *P. hawaiensis*.

		Gryllus bimaculatus				***Oncopeltus fasciatus***				***Parhyale hawaiensis***			
Amino Acid	Codon	Mean High	SE High	Mean Low	SE low	Mean High	SE high	Mean Low	SE low	Mean High	SE high	Mean Low	SE low
Ala	GCT	1.793	0.019	1.513	0.027	2.016	0.022	1.645	0.026	1.373	0.026	1.324	0.021
Ala	GCC	0.565	0.013	0.625	0.018	0.701	0.015	0.782	0.020	1.052	0.025	1.023	0.020
Ala	GCA	1.410	0.019	1.465	0.027	1.107	0.017	1.228	0.023	1.058	0.025	1.046	0.020
Ala	GCG	0.232	0.009	0.355	0.016	0.176	0.009	0.277	0.015	0.517	0.020	0.596	0.015
Arg	CGT	1.278	0.026	1.000	0.028	0.670	0.020	0.572	0.024	0.967	0.035	0.906	0.024
Arg	CGC	0.593	0.020	0.579	0.023	0.310	0.015	0.412	0.019	0.914	0.033	0.916	0.024
Arg	CGA	1.089	0.023	1.008	0.029	0.794	0.023	0.814	0.025	0.841	0.037	0.892	0.023
Arg	CGG	0.390	0.015	0.360	0.017	0.332	0.015	0.379	0.017	0.582	0.027	0.745	0.023
Arg	AGA	1.841	0.030	2.101	0.042	2.298	0.036	2.247	0.039	1.557	0.046	1.435	0.031
Arg	AGG	0.809	0.020	0.828	0.025	1.576	0.033	1.459	0.035	1.139	0.040	1.075	0.025
Asn	AAT	1.414	0.011	1.340	0.014	1.403	0.011	1.276	0.015	0.910	0.020	0.964	0.015
Asn	AAC	0.586	0.011	0.646	0.013	0.597	0.011	0.707	0.015	1.073	0.020	0.994	0.015
Asp	GAT	1.469	0.010	1.336	0.015	1.412	0.011	1.282	0.014	1.048	0.017	0.969	0.015
Asp	GAC	0.531	0.010	0.622	0.014	0.588	0.011	0.701	0.014	0.946	0.017	0.978	0.015
Cys	TGT	1.290	0.021	1.119	0.019	1.252	0.021	1.036	0.021	0.748	0.026	0.915	0.018
Cys	TGC	0.627	0.019	0.660	0.017	0.677	0.020	0.674	0.019	0.904	0.027	0.918	0.018
Gln	CAA	1.082	0.013	1.153	0.016	1.114	0.014	1.051	0.016	0.851	0.019	0.916	0.015
Gln	CAG	0.910	0.013	0.813	0.016	0.880	0.014	0.893	0.016	1.125	0.020	1.047	0.016
Glu	GAA	1.420	0.010	1.357	0.013	1.406	0.010	1.298	0.014	1.078	0.016	1.083	0.014
Glu	GAG	0.580	0.010	0.602	0.012	0.588	0.010	0.680	0.013	0.910	0.016	0.880	0.014
Gly	GGT	1.398	0.021	1.217	0.026	1.353	0.019	1.035	0.022	1.145	0.030	1.124	0.023
Gly	GGC	0.667	0.017	0.685	0.020	0.605	0.016	0.788	0.021	1.193	0.032	1.176	0.024
Gly	GGA	1.510	0.020	1.498	0.026	1.518	0.018	1.545	0.026	1.096	0.033	1.000	0.022
Gly	GGG	0.425	0.012	0.558	0.018	0.524	0.014	0.598	0.018	0.541	0.024	0.668	0.019

His	CAT	1.388	0.016	1.258	0.018	1.307	0.018	1.204	0.019	0.945	0.025	0.931	0.017
His	CAC	0.597	0.015	0.632	0.016	0.661	0.017	0.690	0.018	0.943	0.025	0.979	0.018
Ile	ATT	1.758	0.015	1.447	0.020	1.545	0.014	1.378	0.019	1.156	0.027	1.184	0.019
Ile	ATC	0.469	0.013	0.604	0.017	0.632	0.014	0.665	0.017	1.156	0.027	1.019	0.020
Ile	ATA	0.761	0.015	0.887	0.017	0.823	0.014	0.924	0.016	0.653	0.023	0.765	0.017
Leu	TTA	1.251	0.021	1.286	0.022	1.285	0.024	1.211	0.025	0.580	0.023	0.806	0.019
Leu	TTG	1.647	0.021	1.347	0.022	1.037	0.017	1.020	0.020	1.151	0.029	1.098	0.021
Leu	CTT	1.344	0.018	1.275	0.022	1.807	0.026	1.461	0.022	1.017	0.030	1.012	0.019
Leu	CTC	0.463	0.013	0.680	0.019	0.709	0.019	0.856	0.023	1.207	0.034	1.061	0.020
Leu	CTA	0.472	0.012	0.547	0.014	0.550	0.013	0.662	0.017	0.575	0.026	0.639	0.016
Leu	CTG	0.822	0.016	0.844	0.020	0.613	0.016	0.791	0.021	1.471	0.033	1.383	0.025
Lys	AAA	1.214	0.011	1.247	0.013	1.206	0.010	1.186	0.013	0.947	0.018	1.047	0.015
Lys	AAG	0.786	0.011	0.726	0.013	0.794	0.010	0.797	0.013	1.042	0.018	0.931	0.014
Phe	TTT	1.290	0.014	1.227	0.015	1.304	0.014	1.186	0.017	0.868	0.021	0.944	0.015
Phe	TTC	0.695	0.014	0.711	0.014	0.696	0.014	0.775	0.017	1.102	0.021	1.011	0.015
Pro	CCT	1.746	0.021	1.587	0.028	1.897	0.026	1.703	0.027	1.443	0.033	1.279	0.024
Pro	CCC	0.456	0.014	0.646	0.021	0.510	0.018	0.563	0.019	0.951	0.032	0.887	0.021
Pro	CCA	1.530	0.020	1.391	0.027	1.358	0.020	1.368	0.026	1.013	0.029	1.121	0.022
Pro	CCG	0.268	0.012	0.306	0.016	0.209	0.013	0.333	0.015	0.546	0.023	0.676	0.019
Ser	TCT	1.668	0.024	1.370	0.023	1.909	0.025	1.550	0.026	1.206	0.030	1.191	0.022
Ser	TCC	0.551	0.014	0.751	0.021	0.638	0.019	0.780	0.020	0.965	0.030	0.989	0.020
Ser	TCA	1.459	0.021	1.483	0.025	1.484	0.022	1.360	0.024	1.019	0.031	0.981	0.019
Ser	TCG	0.345	0.014	0.353	0.014	0.205	0.011	0.332	0.015	0.801	0.029	0.711	0.018
Ser	AGT	1.389	0.022	1.364	0.026	1.129	0.022	1.210	0.024	0.951	0.027	1.030	0.020
Ser	AGC	0.589	0.017	0.679	0.019	0.635	0.021	0.766	0.020	1.058	0.033	1.099	0.022
Thr	ACT	1.560	0.020	1.397	0.023	1.811	0.021	1.567	0.027	1.249	0.029	1.215	0.022
Thr	ACC	0.560	0.013	0.665	0.018	0.623	0.017	0.745	0.020	1.052	0.028	0.938	0.020
Thr	ACA	1.554	0.020	1.514	0.023	1.355	0.020	1.388	0.024	0.957	0.026	1.048	0.021
Thr	ACG	0.311	0.014	0.425	0.016	0.186	0.009	0.267	0.013	0.730	0.025	0.783	0.018
Tyr	TAT	1.272	0.015	1.190	0.018	1.289	0.016	1.113	0.017	0.761	0.022	0.867	0.017
Tyr	TAC	0.706	0.015	0.644	0.016	0.685	0.015	0.826	0.017	1.121	0.024	1.035	0.017

Val	GTT	1.581	0.018	1.338	0.022	1.734	0.020	1.472	0.026	1.077	0.025	1.109	0.019
Val	GTC	0.403	0.011	0.544	0.015	0.632	0.013	0.733	0.020	0.957	0.024	0.876	0.018
Val	GTA	0.849	0.014	0.975	0.020	0.875	0.016	0.983	0.022	0.697	0.023	0.818	0.018
Val	GTG	1.167	0.015	1.129	0.021	0.758	0.016	0.800	0.019	1.257	0.026	1.187	0.021

Table S3 The size complexity (S/C) scores per amino acid as per Dufton et al. (1997).

Ala	A	4.76
Arg	R	56.34
Asn	N	33.72
Asp	D	32.72
Cys	C	57.16
Gln	Q	37.48
Glu	E	36.48
Gly	G	1
His	H	58.7
Ile	I	16.04
Leu	L	16.04
Lys	K	30.14
Met	M	64.68
Phe	F	44
Pro	P	31.8
Ser	S	17.86
Thr	T	21.62
Trp	W	73
Tyr	Y	57
Val	V	12.28

Table S4 Functional clustering of the pooled moderate and low expressed CDS (all CDS below the 95th percentile of RPM) for each of three arthropod species under study using their orthologs in the model *D. melanogaster*. The orthologs of CDS below the 95th percentile in expression per species were submitted to gene ontology system DAVID (DUFTON 1997) using identifiers of their *D. melanogaster* orthologs. Functional categories with enrichment values >2.5 are shown. P-values represent a modified Fisher's test, wherein lower values indicate greater enrichment.

Gryllus bimaculatus		***Oncopeltus fasciatus***		***Parhyale hawaiensis***	
**Enrichment Score: 22.76**	P-Value	**Enrichment Score: 17.93**	P-Value	**Enrichment Score: 22.76**	P-value
nucleotide-binding	1.00E-27	nucleotide binding	1.30E-21	nucleotide-binding	1.90E-16
atp-binding	2.90E-27	nucleoside binding	1.50E-19	atp-binding	4.10E-14
purine ribonucleotide binding	2.80E-24	purine nucleotide binding	2.50E-19	purine ribonucleotide binding	1.30E-12
ribonucleotide binding	2.80E-24	purine nucleoside binding	5.70E-19	ribonucleotide binding	8.10E-12
purine nucleotide binding	4.00E-24	adenyl nucleotide binding	3.20E-18	purine nucleotide binding	2.10E-11
nucleoside binding	6.10E-23	ribonucleotide binding	5.40E-18	nucleoside binding	2.10E-11
purine nucleoside binding	6.30E-22	purine ribonucleotide binding	5.40E-18	purine nucleoside binding	1.50E-10
adenyl ribonucleotide binding	1.30E-21	adenyl ribonucleotide binding	3.50E-17	adenyl ribonucleotide binding	2.20E-10
nucleotide binding	1.30E-21	ATP binding	4.70E-17	nucleotide binding	4.20E-10
adenyl nucleotide binding	2.10E-21	**Enrichment Score: 15.66**		adenyl nucleotide binding	5.00E-09
ATP binding	3.90E-21	organelle lumen	2.00E-16	ATP binding	7.20E-09
**Enrichment Score: 8.93**		intracellular organelle lumen	2.00E-16	**Enrichment Score: 8.93**	
endocytosis	2.40E-10	membrane-enclosed lumen	2.80E-16	endocytosis	2.40E-07
membrane invagination	2.40E-10	**Enrichment Score: 12.68**		membrane invagination	6.00E-06
membrane organization	2.60E-08	membrane organization	8.10E-14	membrane organization	9.80E-06
**Enrichment Score: 8.51**		endocytosis	3.40E-13	**Enrichment Score: 8.51**	5.20E-04

intracellular organelle lumen	3.00E-09	membrane invagination	3.40E-13	intracellular organelle lumen	
organelle lumen	3.00E-09	**Enrichment Score: 7.58**		organelle lumen	4.50E-06
membrane-enclosed lumen	3.30E-09	protein complex biogenesis	2.20E-08	membrane-enclosed lumen	4.50E-06
**Enrichment Score: 7.29**		protein complex assembly	2.20E-08	**Enrichment Score: 7.29**	7.00E-06
serine/threonine-protein kinase	1.20E-10	macromolecular complex assembly	3.60E-08	serine/threonine-protein kinase	1.00E-04
protein amino acid phosphorylation	3.90E-09	**Enrichment Score: 7.5**		protein amino acid phosphorylation	1.90E-04
protein kinase activity	1.80E-08	cellular macromolecule catabolic process	3.60E-10	protein kinase activity	3.20E-04
Protein kinase	4.5E-05	macromolecule catabolic process	4.70E-09	Protein kinase	3.90E-04
protein serine/threonine kinase activity	2.00E-08	protein catabolic process	7.50E-08	protein serine/threonine kinase activity	4.90E-04
Protein kinase	5.5E-02	modification-dependent protein catabolic process	9.90E-08	Protein kinase	6.00E-04
Serine/threonine protein kinase	5.5E-02	modification-dependent macromolecule catabolic process	1.30E-07	Serine/threonine protein kinase	
Serine/threonine protein kinase-related	4.40E-06	proteolysis involved in cellular protein catabolic process	1.40E-07	Serine/threonine protein kinase-related	2.20E-08
**Enrichment Score: 6.76**		cellular protein catabolic process	1.40E-07	**Enrichment Score: 6.76**	2.30E-06
phosphorylation	1.70E-07	**Enrichment Score: 6.49**		phosphorylation	3.10E-06
phosphorus metabolic process	1.70E-07	helicase	4.50E-09	phosphorus metabolic process	1.10E-05
phosphate metabolic process	1.70E-07	purine NTP-dependent helicase activity	8.30E-08	phosphate metabolic process	1.10E-05
**Enrichment Score: 6.59**		ATP-dependent helicase activity	8.30E-08	**Enrichment Score: 6.59**	2.00E-04
wd repeat	1.50E-09	DEXDc	1.80E-07	wd repeat	1.00E-03
WD40	2.30E-07	HELICc	2.80E-07	WD40	3.50E-03
WD40/YVTN repeat-like	2.50E-07	DEAD-like helicase	1.60E-06	WD40/YVTN repeat-like	4.30E-03
WD40 repeat	3.80E-07	DNA/RNA helicase	2.50E-06	WD40 repeat	5.70E-03

WD40 repeat		Helicase	2.50E -06	WD40 repeat	2.10E-02
WD40 repeat	2.40E-03	**Enrichment Score: 6.06**		**WD40 repeat**	
WD40 repeat 2	2.90E-06	organellar ribosome	9.20E-09	WD40 repeat 2	1.00E-04
**Enrichment Score: 6.31**		mitochondrial ribosome	9.20E-09	**Enrichment Score: 6.31**	1.00E-04
helicase	3.30E-08	ribosomal subunit	7.80E-03	helicase	2.20E-04
HELICc	4.20E-07	**Enrichment Score: 5.96**		HELICc	6.70E-04
DNA/RNA helicase	1.40E-01	phosphorus metabolic process	1.00E-06	**DNA/RNA helicase**	
DEXDc	9.10E-07	phosphate metabolic process	1.00E-06	DEXDc	1.30E-05
DEAD-like helicase	1.80E-01	phosphorylation	1.30E-06	DEAD-like helicase	1.40E-05
Helicase	2.50E-01	**Enrichment Score: 5.9**		Helicase	8.00E-05
**Enrichment Score: 5.79**		cellular protein localization	6.00E-07	**Enrichment Score: 5.79**	1.30E-04
cellular macromolecule catabolic process	2.90E-08	cellular macromolecule localization	1.80E-06	cellular macromolecule catabolic process	1.30E-04
protein catabolic process	3.20E-07	intracellular protein transport	1.90E-06	protein catabolic process	1.70E-04
modification-dependent protein catabolic process	4.50E-06	**Enrichment Score: 5.85**		modification-dependent protein catabolic process	1.90E-04
modification-dependent macromolecule catabolic process	5.60E-06	transcription initiation from RNA polymerase II promoter	3.50E-07	modification-dependent macromolecule catabolic process	2.90E-04
proteolysis involved in cellular protein catabolic process	8.80E-06	general RNA polymerase II transcription factor activity	2.50E-06	proteolysis involved in cellular protein catabolic process	1.30E-01
cellular protein catabolic process	8.80E-06	transcription initiation	3.30E-06	cellular protein catabolic process	7.40E-01
**Enrichment Score: 5.71**		**Enrichment Score: 5.18**		**Enrichment Score: 5.71**	
Tetratricopeptide TPR-1	1.50E-07	PHD	1.70E -07	Tetratricopeptide TPR-1	1.10E-04
TPR	3.20E-06	Zinc finger	9.50E-07	TPR	1.10E-04
Tetratricopeptide repeat	3.80E-06	Zinc finger	4.60E-05	Tetratricopeptide repeat	1.50E-04
Tetratricopeptide region	7.60E-06	Zinc finger		Tetratricopeptide region	3.30E-04

**Enrichment Score: 5.45**		**Enrichment Score: 4.75**		**Enrichment Score: 5.45**	2.80E-03
RNA transport	6.60E-07	DNA-directed RNA polymerase activity	1.00E-06	RNA transport	4.30E-03
nucleic acid transport	6.60E-07	RNA polymerase activity	1.00E-06	**nucleic acid transport**	
establishment of RNA localization	1.00E-06	RNA polymerase	5.60E-03	establishment of RNA localization	6.00E-06
nucleobase	1.90E-01	**Enrichment Score: 4.74**		nucleobase	1.30E-04
RNA localization	7.70E-04	ubiquitin-protein ligase activity	6.50E-06	RNA localization	2.00E-04
**Enrichment Score: 5.01**		small conjugating protein ligase activity	7.10E-06	**Enrichment Score: 5.01**	2.70E-03
nucleoside-triphosphatase regulator activity	4.40E-06	acid-amino acid ligase activity	1.30E-04	nucleoside-triphosphatase regulator activity	3.10E-03
GTPase regulator activity	4.80E-06	**Enrichment Score: 4.39**		GTPase regulator activity	3.50E-03
small GTPase regulator activity	4.50E-05	mitochondrial large ribosomal subunit	1.90E-06	small GTPase regulator activity	8.30E-03
**Enrichment Score: 4.11**		organellar large ribosomal subunit	1.90E-06	**Enrichment Score: 4.11**	
PHD	3.30E-05	large ribosomal subunit	2.00E-02	PHD	2.80E-05
Zinc finger	5.30E-01	**Enrichment Score: 4.25**		Zinc finger	1.50E-03
Zinc finger	8.00E-01	maintenance of protein location	1.20E-05	Zinc finger	4.30E-02
Zinc finger		maintenance of location	3.10E-05	**Zinc finger**	
**Enrichment Score: 4.07**		maintenance of protein location in cell	1.00E-04	**Enrichment Score: 4.07**	4.30E-05
cell-cell junction organization	1.60E-05	maintenance of location in cell	2.70E-04	cell-cell junction organization	2.10E-04
cell junction organization	2.80E-05	**Enrichment Score: 4.11**		cell junction organization	1.20E-02
apical junction assembly	9.10E-05	cation binding	3.60E-05	apical junction assembly	6.80E-02
cell-cell junction assembly	2.60E-04	metal ion binding	3.60E-05	cell-cell junction assembly	
cell junction assembly	4.30E-04	ion binding	6.00E-05	cell junction assembly	7.60E-05
**Enrichment Score: 3.86**		transition metal ion binding	4.40E-04	**Enrichment Score: 3.86**	4.70E-04

DNA-directed RNA polymerase complex	6.50E-05	**Enrichment Score: 4.05**		DNA-directed RNA polymerase complex	1.10E-03
nuclear DNA-directed RNA polymerase complex	6.50E-05	protein amino acid phosphorylation	8.30E-06	nuclear DNA-directed RNA polymerase complex	2.40E-03
RNA polymerase complex	6.50E-05	Protein kinase	2.70E-04	RNA polymerase complex	3.90E-03
RNA polymerase activity	1.70E-04	protein kinase activity	3.10E-04	RNA polymerase activity	5.20E-03
DNA-directed RNA polymerase activity	1.70E-04	**Enrichment Score: 3.85**		DNA-directed RNA polymerase activity	6.90E-03
RNA polymerase	8.20E-04	generation of a signal involved in cell-cell signaling	2.30E-05	RNA polymerase	2.90E-02
**Enrichment Score: 3.36**		secretion by cell	4.10E-05	**Enrichment Score: 3.36**	
aging	4.30E-04	neurotransmitter secretion	4.20E-05	aging	2.50E-07
determination of adult life span	4.30E-04	regulation of neurotransmitter levels	1.40E-04	determination of adult life span	3.10E-05
multicellular organismal aging	4.30E-04	secretion	2.10E-04	multicellular organismal aging	1.10E-04
Enrichment Score: 3.32		neurotransmitter transport	6.40E-03	**Enrichment Score: 3.32**	1.30E-04
sh3 domain	7.50E-05	**Enrichment Score: 3.7**		sh3 domain	4.80E-04
SH3	1.10E-03	kelch repeat	6.90E-05	SH3	1.00E-03
Src homology-3 domain	1.30E-03	Kelch	1.70E -04	Src homology-3 domain	1.30E-03
**Enrichment Score: 3.24**		Kelch-type beta propeller	3.70E-04	Enrichment Score: 3.24	3.10E-03
kelch repeat	2.60E-04	Kelch repeat type 1	3.70E-04	kelch repeat	1.20E-02
Kelch	7.20E-04	**Enrichment Score: 3.66**		Kelch	8.60E-02
Kelch repeat type 1	7.70E-04	wd repeat	4.60E-07	Kelch repeat type 1	1.10E-01
Kelch-type beta propeller	7.70E-04	WD40	6.10E -05	Kelch-type beta propeller	1.70E-01
**Enrichment Score: 3.21**		WD40/YVTN repeat-like	1.90E-04	Enrichment Score: 3.21	1.80E-01
gtp-binding	2.70E-04	WD40 repeat	6.30E-04	gtp-binding	1.90E-01
guanyl ribonucleotide binding	7.40E-04	WD40 repeat	7.90E-04	guanyl ribonucleotide binding	

GTP binding	8.20E-04	WD40 repeat	1.70E-03	GTP binding	2.50E-05
guanyl nucleotide binding	8.70E-04	WD40 repeat 2	5.40E-03	guanyl nucleotide binding	4.30E-05
**Enrichment Score: 3.1**		**Enrichment Score: 3.65**		**Enrichment Score: 3.1**	8.00E-04
ank repeat	1.80E-04	ank repeat	4.20E-05	ank repeat	3.70E-03
ANK	1.50E-03	ANK	2.30E -04	ANK	1.00E-02
Ankyrin	1.80E-03	Ankyrin	1.10E -03	Ankyrin	1.50E-02
**Enrichment Score: 3.07**		**Enrichment Score: 3.64**		**Enrichment Score: 3.07**	3.00E-02
epithelium development	3.40E-04	nuclear DNA-directed RNA polymerase complex	7.80E-05	epithelium development	RT
morphogenesis of an epithelium	7.40E-04	DNA-directed RNA polymerase complex	7.80E-05	morphogenesis of an epithelium	5.20E-02
tissue morphogenesis	2.50E-03	RNA polymerase complex	7.80E-05	tissue morphogenesis	1.70E-01
**Enrichment Score: 2.83**		RNA polymerase	5.60E-03	**Enrichment Score: 2.83**	
glycerophospholipid metabolic process	3.00E-04	**Enrichment Score: 3.25**		glycerophospholipid metabolic process	8.70E-06
glycerolipid metabolic process	6.70E-04	serine/threonine-protein kinase	1.00E-04	glycerolipid metabolic process	8.50E-05
phospholipid metabolic process	3.00E-03	Serine/threonine protein kinase-related	4.50E-04	phospholipid metabolic process	5.90E-04
organophosphate metabolic process	8.20E-03	protein serine/threonine kinase activity	8.10E-04	organophosphate metabolic process	5.90E-04
**Enrichment Score: 2.71**		Serine/threonine protein kinase	2.80E-03	**Enrichment Score: 2.71**	6.00E-04
RNA-dependent ATPase activity	1.50E-03	**Enrichment Score: 3.22**		RNA-dependent ATPase activity	7.40E-04
ATP-dependent RNA helicase activity	1.50E-03	ribosomal protein	3.40E-06	ATP-dependent RNA helicase activity	2.90E-03
RNA helicase activity	3.40E-03	structural constituent of ribosome	1.20E-03	RNA helicase activity	3.40E-03
**Enrichment Score: 2.62**		ribosome	4.30E-03	**Enrichment Score: 2.62**	
ubiquitin-protein ligase activity	3.50E-04	ribosomal subunit	7.80E-03	ubiquitin-protein ligase activity	6.30E-03
small conjugating protein ligase activity	1.50E-03	**Enrichment Score: 3.22**		small conjugating protein ligase activity	1.50E-02

ligase activity	5.40E-02	aging	6.00E -04	ligase activity	1.60E-02
acid-amino acid ligase activity	1.00E-02	determination of adult life span	6.00E-04	acid-amino acid ligase activity	1.60E-02
**Enrichment Score: 2.59**		multicellular organismal aging	6.00E-04	**Enrichment Score: 2.59**	RT
metal ion binding	1.70E-03	**Enrichment Score: 3.16**		metal ion binding	2.50E-02
cation binding	2.40E-03	transmission of nerve impulse	5.50E-04	cation binding	9.80E-01
ion binding	2.90E-03	synaptic transmission	5.60E-04	ion binding	4.20E-01
transition metal ion binding	3.80E-03	cell-cell signaling	1.00E-03	transition metal ion binding	5.30E-01
**Enrichment Score: 2.53**		**Enrichment Score: 3.14**		**Enrichment Score: 2.53**	
mitochondrial small ribosomal subunit	3.50E-04	glycerophospholipid metabolic process	1.70E-04	mitochondrial small ribosomal subunit	2.20E-08
organellar small ribosomal subunit	3.50E-04	glycerolipid metabolic process	4.60E-04	organellar small ribosomal subunit	2.60E-05
small ribosomal subunit	2.00E-01	phosphoinositide metabolic process	5.00E-03	small ribosomal subunit	6.50E-05
		**Enrichment Score: 2.86**			
		nucleoside-triphosphatase regulator activity	7.50E-04		
		GTPase regulator activity	1.50E-03		
		small GTPase regulator activity	2.40E-03		
		**Enrichment Score: 2.83**			
		regulation of actin polymerization or depolymerization	8.60E-04		
		regulation of actin filament length	8.60E-04		
		regulation of actin filament polymerization	4.50E-03		
		**Enrichment Score: 2.78**			
		apicolateral plasma membrane	3.90E-04		
		apical junction complex	1.90E-03		

cell-cell junction	6.10E-03
**Enrichment Score: 2.72**	
RNA helicase activity	5.20E-04
ATP-dependent RNA helicase activity	3.70E-03
RNA-dependent ATPase activity	3.70E-03
**Enrichment Score: 2.64**	
mitochondrial electron transport	6.10E-04
oxidoreductase activity	7.50E-04
NADH dehydrogenase activity	1.40E-03
NADH dehydrogenase (quinone) activity	2.50E-03
oxidoreductase activity	
NADH dehydrogenase (ubiquinone) activity	2.50E-03
NADH dehydrogenase complex	5.40E-03
respiratory chain complex I	5.40E-03
mitochondrial respiratory chain complex I	5.40E-03
Enrichment Score: 2.59	
establishment of RNA localization	1.50E-03
RNA transport	2.70E-03
nucleic acid transport	2.70E-03
nucleobase	
**Enrichment Score: 2.56**	
apical junction assembly	1.20E-03

cell-cell junction assembly	2.80E-03
cell-cell junction organization	2.90E-03
cell junction organization	4.10E-03
cell junction assembly	4.20E-03
**Enrichment Score: 2.55**	
organellar small ribosomal subunit	4.70E-04
mitochondrial small ribosomal subunit	4.70E-04
small ribosomal subunit	1.00E-01
**Enrichment Score: 2.53**	
Spectrin repeat	1.80E-03
SPEC	2.90E-03
Spectrin/alpha-actinin	4.70E-03
**Enrichment Score: 2.53**	
nuclear division	2.30E-03
organelle fission	2.90E-03
mitosis	2.90E-03
M phase of mitotic cell cycle	4.00E-03
**Enrichment Score: 2.5**	
3'-5'-exoribonuclease activity	2.40E-03
exoribonuclease activity	3.60E-03
exoribonuclease activity	3.60E-03

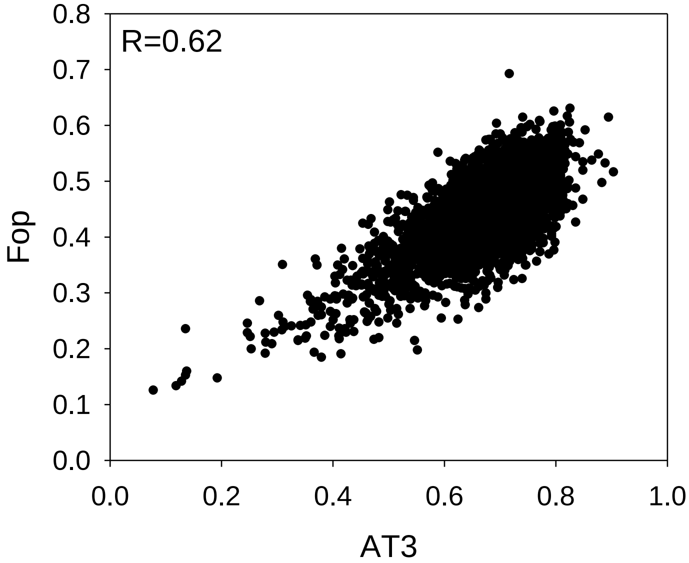

A. *Gryllus bimaculatus*

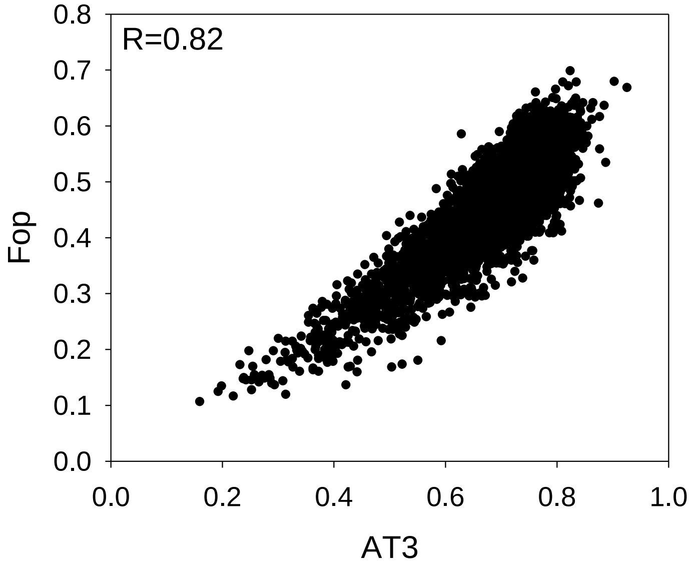

B. *Oncopeltus fasciatus*

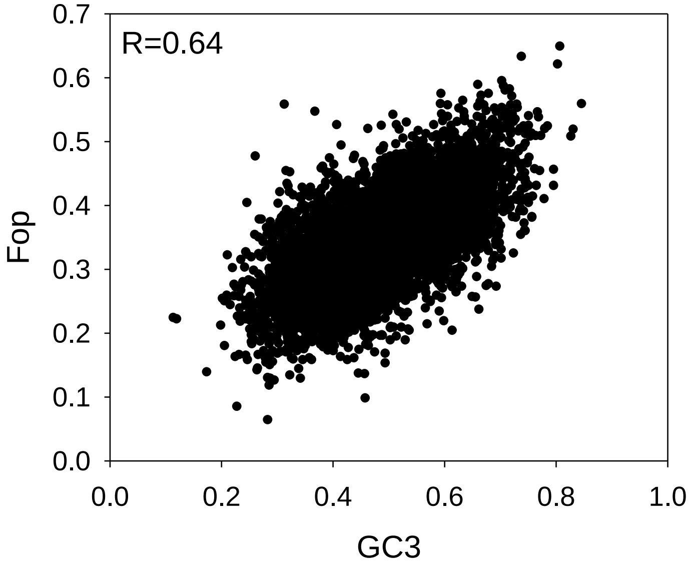

C. *Parhyale hawaiensis*

Figure S1 The Spearman rank correlation A) AT3 and Fop for *G. bimaculatus*. B) AT3 and Fop for *O. fasciatus*. C) GC3 and Fop for *P. hawaiensis*. $P < 10^{-15}$ for all correlations. Pearson correlations yielded nearly identical results (not shown).

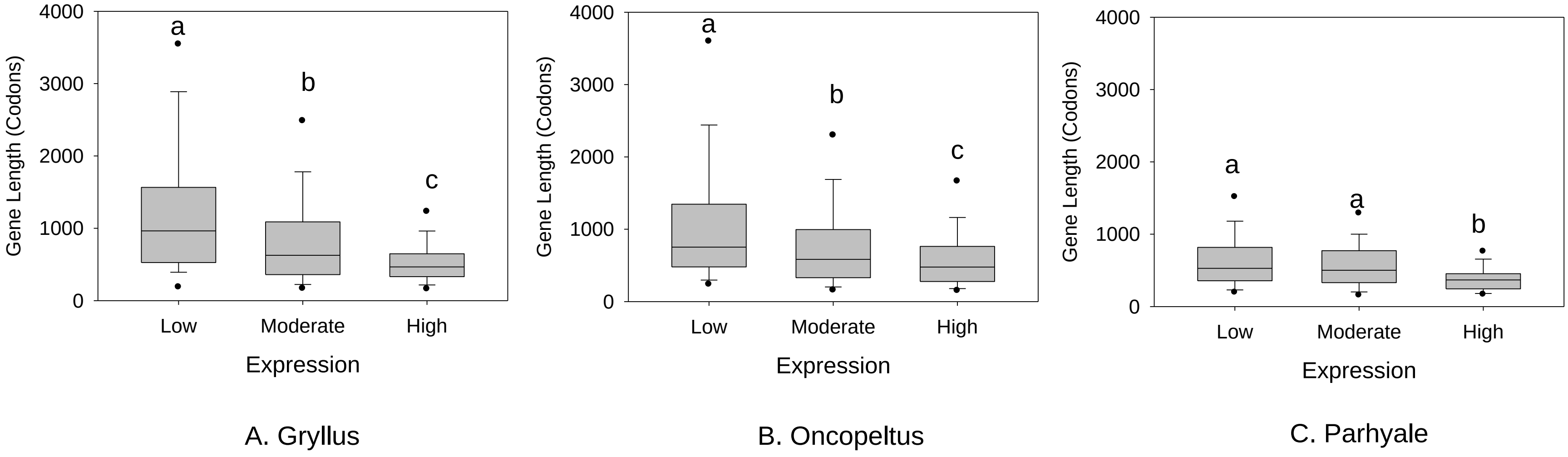

Figure S2 Bar and whisker plots of CDS length (number of codons) of *D. melanogaste*r orthologs to CDS with low, moderate and high expression in A) *G. bimaculatus;* B) *O. fasciatus*; and C) *P. hawaiensis*. P-values of Ranked-ANOVA $<3.9X10^{-9}$ for each figure. Different letters in each figure indicate paired differences using Dunn's contrast (P<0.05).

File S1

Supporting Results File 1

In the analysis of RSCU described in our Results section, we chose to use RPM to measure expression rather than reads per kilobase million (RPKM). This is because while the CDS lengths of assembled transcripts for *G. bimaculatus* and *O. fasciatus* were generated using both normalized and non-normalized libraries (EWEN-CAMPEN *et al.* 2011; ZENG *et al.* 2013; ZENG and EXTAVOUR 2012), we quantified expression levels for this study using solely non-normalized libraries, which most directly correlates to expression level (OSHLACK *et al.* 2010). We anticipated that using RPKM rather than RPM would thus likely skew some highly expressed genes towards lower values by underestimating their expression levels, because a transcript segment present in normalized libraries may contribute to transcript assembly by extending gene length, but not have hits in the non-normalized dataset. Indeed, this prediction was borne out when we determined optimal codons using RPKM. Overall we obtained similar results to those obtained with the RPM method (Table 1). However, for *G. bimaculatus* we identified only 10 of the original 17 optimal codons, as seven became non-significant (importantly, 17 of the 17 had positive ΔRSCU using RPM and RPKM, consistent with optimization detected using both methods), whilst we identified 12 of the original 16 optimal codons for *O. fasciatus* (16 of the 16 optimal codons had positive ΔRSCU using RPM and RPKM) (Table 1). For *P. hawaiensis*, we found the exact same 13 optimal codons as those originally identified with the RPM method (Table 1), consistent with the fact that normalized libraries were not used for the transcriptome assembly in this species (ZENG *et al.* 2011). Thus, there is moderate variation in P-values among results obtained with the RPM and RPKM methods. Collectively, from these data we conclude that the only potential effect of using RPM (as opposed to RPKM) to define our 5% most highly or lowly expressed gene lists would possibly be an over-representation of highly expressed long CDS (relative to highly expressed shorter CDS), due to more read matches to longer CDS. This could only affect our results if both the following were true: 1) longer CDS exhibited elevated levels of AT3 or GC3 for reasons other than selection on codon usage (e.g. mutational bias), and 2) the high expression dataset consisted mostly of long genes. However, we examined these possibilities empirically and found that neither of these factors play a role here. To test mutation, we examined the lowest expression 5% RPM category, where selection effects on codon usage should be minimal or

absent (and thus AT variation should be explained solely by mutation): we found no correlation between CDS length (that predicted using transcript read assembly) and AT3 content for *G. bimaculatus* (Spearmans Correlation P=0.37) or *O. fasciatus* (P=0.85), indicating no evidence of a relationship between mutational bias and assembled CDS length. In terms of CDS length, we found the CDS sequences assembled in the 5% highest RPM class consisted of a range of short and long lengths (for example, for *G. bimaculatus* CDS ranged between 102 codons to 2039, with a mean of 466±16.7), and thus spans a range of lengths. Taken together, we conclude the RPM values (as compared to RPKM) provide the most rigorous method for identification of optimal codons in these datasets, as indicated by a strong correspondence to results from RPKM, but with stronger P-values.

It is worth noting that our results showing Fop increases with expression level in Figure 2 were the same regardless of whether we used the RPM or RPKM list of optimal codons. For instance, using the RPKM list for *G. bimaculatus* and for *O. fasciatus*, Fop was found to increase from the low ($Mean_{G.\ bimaculatus}$=0.352±0.006, $Mean_{O.\ fasciatus}$,=0.336±0.005), to the moderate $Mean_{G.\ bimaculatus}$ 0.374±0.001, $Mean_{O.\ fasciatus}$,=0.373±0.001) to the high expression ($Mean_{G.\ bimaculatus}$=0.403±0.003, $Mean_{O.\ fasciatus}$,=0.391±0.004) class for *G. bimaculatus* and for *O. fasciatus* (Ranked ANOVA P<0.001, Dunns Paired test P<0.05 for each contrast per species).

File S2

Supporting Results File 2

We identified orthologs of the 87 ribosomal protein genes (RPGs) in *D. melanogaster* (http://ribosome.med.miyazaki-u.ac.jp/) using BLASTX to the reduced CDS list (without isoforms, and with ORF with a start codon) in *G. bimaculatus*, *O. fasciatus*, and of *P. hawaiensis*. We then concatenated CDS for the RPGs dataset and for the lowly expressed CDS per species, and determined $\Delta RSCU_{RPGs}$= RSCU $_{RPGs}$ – $RSCU_{CDS\ with\ Lowest\ 5\%\ Expression}$. Whilst signals were weakened as compared to the full high expression gene set used in Table 1, especially for two-fold synonymous sites in *G. bimaculatus* and *O. fasciatus* (likely due to the small dataset size of RPGs and low selection at two-fold sites (Table 1)), the results from amino acids with three or more amino acids that exhibit the greatest selection on codon usage (Table 1), support the presence of AT3 optimal codons in these organisms. For instance, for *G. bimaculatus* and for *O. fasciatus*, the optimal codon for nearly all of nine amino acids having three or more synonymous codons in Table 1, yielded a positive $\Delta RSCU_{RPGs}$ (values between +0.12 and +0.73), thus confirming their enhanced usage in highly expressed genes (RPGs). An exception was Arg in *O. fasciatus*, where the optimal codon identified in Table 1 was CGT, even though AGG had a larger ΔRSCU (non-significant); using RPGs, AGG had fourfold higher $\Delta RSCU_{RPGs}$,. A second exception was Pro for *G. bimaculatus* where the optimal codon using RPGs was CCT rather than CCA. For *P. hawaiensis*, 11 of the 13 optimal codons in Table 1 were also identified using $\Delta RSCU_{RPGs}$,. A switch was observed for two amino acids: GGA to GGT for Gly and TCG to TCC for Ser, each staying within the AT3 or GC3 codon family, respectively. Notably, additional amino acids had codons with substantial positive $\Delta RSCU_{RPGs}$ for *P. hawaiensis* and might be putative optimal codons, such as Arg (both CGC and CGT), Cys (TGC), His (CAC) and Glu (GAG). Thus, GC3 codons might be favored across a wider spectrum of amino acids than reported in Table 1 ($\Delta RSCU_{RPGs}$ values ranged from +0.20 to +0.73). We therefore consider the lists in Table 1 for *P. hawaiensis* spanning 13 amino acids to be conservative. Future genomic sequence data will help resolve these variations. Together, $\Delta RSCU_{RPGs}$ analysis concurs with prevalence of AT3 optimal codons in *G. bimaculatus* and *O. fasciatus*, and GC3 optimal codons in *P. hawaiensis*.

File S3

Supporting Materials and Methods

Transcriptome data from RNA-seq for oogenesis and embryogenesis of *G. bimaculatus* (ZENG *et al.* 2013) *O. fasciatus* (EWEN-CAMPEN *et al.* 2011) and *P. hawaiensis* (ZENG *et al.* 2011) were obtained from ASGARD as shown in supplementary Table S1. For each species, we divided the CDS list into two categories: those with isoforms and those without isoforms. The latter class was used for our analyses and to map reads; this allows certainty when matching reads, as isoforms from a single gene can match the same read. In turn, for this reduced CDS set with no isoforms, we extracted the open reading frame (ORF) using ORF Predictor (http://proteomics.ysu.edu/tools/OrfPredictor.html). The final CDS set per species was used to study gene expression profiles, and to identify the sets of the 5% of most highly and lowly expressed genes, which was then used to reveal their optimal codon lists.

Expression level was measured based on the number of hits per CDS for genes without isoforms using MEGABLAST. For each read, the CDS with the greatest percent identity was taken as the match, with a cutoff of >95% identity. Each read matched only one CDS. Expression levels per CDS were calculated by scoring the number of reads mapped from each non-normalized library to the CDS list per species for all genes without isoforms (supplementary Table S1), and was standardized as Reads per million (RPM) = Number of matching reads/Total number of reads matching a CDS X 1,000,000. Reads per kilobase million (RPKM) was calculated as RPM/CDS length X 1,000. Fop was determined using Codon W (Peden, http://codonw.sourceforge.net/). Ribosomal protein genes were identified using BLASTX to query the *D. melanogaster* RPG list http://ribosome.med.miyazaki-u.ac.jp/). Orthologs between *G. bimaculatus*, *O. fasciatus* and *P. hawaiensis* and *D. melanogaster* were also identified using BLASTX, with the latter taxon used as the protein sequence database using the longest CDS per gene. Gene ontology was assessed using DAVID Bioinformatics Resources 6.7 (HUANG DA *et al.* 2009a; HUANG DA *et al.* 2009b). Statistical analysis was conducted using SigmaStat 3.5 (http://www.systat.com).

Supporting Information References

DUFTON, M. J., 1997 Genetic code synonym quotas and amino acid complexity: cutting the cost of proteins? Journal of theoretical biology **187:** 165-173.

EWEN-CAMPEN, B., N. SHANER, K. A. PANFILIO, Y. SUZUKI, S. ROTH *et al.*, 2011 The maternal and embryonic transcriptome of the milkweed bug *Oncopeltus fasciatus*. BMC Genomics **12:** 61.

HUANG DA, W., B. T. SHERMAN and R. A. LEMPICKI, 2009a Bioinformatics enrichment tools: paths toward the comprehensive functional analysis of large gene lists. Nucleic acids research **37:** 1-13.

HUANG DA, W., B. T. SHERMAN and R. A. LEMPICKI, 2009b Systematic and integrative analysis of large gene lists using DAVID bioinformatics resources. Nature protocols **4:** 44-57.

MIN, X. J., G. BUTLER, R. STORMS and A. TSANG, 2005 OrfPredictor: predicting protein-coding regions in EST-derived sequences. Nucleic acids research **33:** W677-680.

OSHLACK, A., M. D. ROBINSON and M. D. YOUNG, 2010 From RNA-seq reads to differential expression results. Genome biology **11:** 220.

ZENG, V., B. EWEN-CAMPEN, H. W. HORCH, S. ROTH, T. MITO *et al.*, 2013 Developmental gene discovery in a hemimetabolous insect: *de novo* assembly and annotation of a transcriptome for the cricket *Gryllus bimaculatus*. PLoS ONE **8:** e61479.

ZENG, V., and C. G. EXTAVOUR, 2012 ASGARD: an open-access database of annotated transcriptomes for emerging model arthropod species. Database **2012:** bas048.

ZENG, V., K. E. VILLANUEVA, B. EWEN-CAMPEN, F. ALWES, W. E. BROWNE *et al.*, 2011 *De novo* assembly and characterization of a maternal and developmental transcriptome for the emerging model crustacean *Parhyale hawaiensis*. BMC Genomics **12:** 581.
