# Supplementary material for: Codon and Amino Acid Usage Are Shaped by Selection Across Divergent Model Organisms of the Pancrustacea
Source: G3 (Bethesda). 2015 Sep 17;5(11):2307–21. doi: 10.1534/g3.115.021402 (PMC4632051; doi:10.1534/g3.115.021402)
Supplement: Supporting Information [file supp_g3.115.021402_TableS1.pdf]

Table S1 Transcript datasets used in the present study. All data per species were used for assembly as described in ASGARD (Ewen-Campen et al. 2011; Zeng et al. 2011; Zeng and Extavour 2012; Zeng et al. 2013). Datasets in bold were used for expression analysis.

	Tissue Type	Sequencing Mode	Sample ID (No. in cited Reference/No. in NCBI)	Library Normalized	No. Reads
G. bimaculatus	Embryos	GS-FLX	SRX023830/SRR060814	Yes	78,936
	Ovaries	GS-FLX	SRX023831/SRR060815	Yes	67,353
	**Pooled Ovaries and Embryos**	**GS_FLX Titanium**	SRX023832/SRR060816	No	4,102,057
				Total	4,248,346
O. fasciatus	**Pooled Ovaries and Embryos**	**GS_FLX Titanium**	SRX022014/ SRR057573	No	1,293,320
	Pooled Ovaries and Embryos	GS_FLX Titanium	SRX022013/ SRR057572	Yes	656, 783
	Embryos	GS-FLX	SRX022012/ SRR057571	Yes	71,912
	Ovaries	GS-FLX	SRX022011/ SRR057570	Yes	65,395
				Total	2,087,410
P. hawaiensis	**Pooled Ovaries and Embryos**	**GS_FLX Titanium**	SRX0238929/SRR060813	No	3,172,925
