# Supplementary material for: Codon and Amino Acid Usage Are Shaped by Selection Across Divergent Model Organisms of the Pancrustacea
Source: G3 (Bethesda). 2015 Sep 17;5(11):2307–21. doi: 10.1534/g3.115.021402 (PMC4632051; doi:10.1534/g3.115.021402)
Supplement: Supporting Information [file supp_g3.115.021402_TableS2.pdf]

Table S2 The mean RSCU and standard errors (SE) for highly and lowly expressed genes in *G. bimaculatus*, *O. fasciatus* and *P. hawaiensis*.

		Gryllus bimaculatus				*Oncopeltus fasciatus*				*Parhyale hawaiensis*			
Amino Acid	Codo n	Mean High	SE High	Mean Low	SE low	Mean High	SE high	Mean Low	SE low	Mean High	SE high	Mean Low	SE low
Ala	GCT	1.793	0.019	1.513	0.027	2.016	0.022	1.645	0.026	1.373	0.026	1.324	0.021
Ala	GCC	0.565	0.013	0.625	0.018	0.701	0.015	0.782	0.020	1.052	0.025	1.023	0.020
Ala	GCA	1.410	0.019	1.465	0.027	1.107	0.017	1.228	0.023	1.058	0.025	1.046	0.020
Ala	GCG	0.232	0.009	0.355	0.016	0.176	0.009	0.277	0.015	0.517	0.020	0.596	0.015
Arg	CGT	1.278	0.026	1.000	0.028	0.670	0.020	0.572	0.024	0.967	0.035	0.906	0.024
Arg	CGC	0.593	0.020	0.579	0.023	0.310	0.015	0.412	0.019	0.914	0.033	0.916	0.024
Arg	CGA	1.089	0.023	1.008	0.029	0.794	0.023	0.814	0.025	0.841	0.037	0.892	0.023
Arg	CGG	0.390	0.015	0.360	0.017	0.332	0.015	0.379	0.017	0.582	0.027	0.745	0.023
Arg	AGA	1.841	0.030	2.101	0.042	2.298	0.036	2.247	0.039	1.557	0.046	1.435	0.031
Arg	AGG	0.809	0.020	0.828	0.025	1.576	0.033	1.459	0.035	1.139	0.040	1.075	0.025
Asn	AAT	1.414	0.011	1.340	0.014	1.403	0.011	1.276	0.015	0.910	0.020	0.964	0.015
Asn	AAC	0.586	0.011	0.646	0.013	0.597	0.011	0.707	0.015	1.073	0.020	0.994	0.015
Asp	GAT	1.469	0.010	1.336	0.015	1.412	0.011	1.282	0.014	1.048	0.017	0.969	0.015
Asp	GAC	0.531	0.010	0.622	0.014	0.588	0.011	0.701	0.014	0.946	0.017	0.978	0.015
Cys	TGT	1.290	0.021	1.119	0.019	1.252	0.021	1.036	0.021	0.748	0.026	0.915	0.018
Cys	TGC	0.627	0.019	0.660	0.017	0.677	0.020	0.674	0.019	0.904	0.027	0.918	0.018
Gln	CAA	1.082	0.013	1.153	0.016	1.114	0.014	1.051	0.016	0.851	0.019	0.916	0.015
Gln	CAG	0.910	0.013	0.813	0.016	0.880	0.014	0.893	0.016	1.125	0.020	1.047	0.016
Glu	GAA	1.420	0.010	1.357	0.013	1.406	0.010	1.298	0.014	1.078	0.016	1.083	0.014
Glu	GAG	0.580	0.010	0.602	0.012	0.588	0.010	0.680	0.013	0.910	0.016	0.880	0.014
Gly	GGT	1.398	0.021	1.217	0.026	1.353	0.019	1.035	0.022	1.145	0.030	1.124	0.023
Gly	GGC	0.667	0.017	0.685	0.020	0.605	0.016	0.788	0.021	1.193	0.032	1.176	0.024
Gly	GGA	1.510	0.020	1.498	0.026	1.518	0.018	1.545	0.026	1.096	0.033	1.000	0.022
Gly	GGG	0.425	0.012	0.558	0.018	0.524	0.014	0.598	0.018	0.541	0.024	0.668	0.019

His	CAT	1.388	0.016	1.258	0.018	1.307	0.018	1.204	0.019	0.945	0.025	0.931	0.017
His	CAC	0.597	0.015	0.632	0.016	0.661	0.017	0.690	0.018	0.943	0.025	0.979	0.018
Ile	ATT	1.758	0.015	1.447	0.020	1.545	0.014	1.378	0.019	1.156	0.027	1.184	0.019
Ile	ATC	0.469	0.013	0.604	0.017	0.632	0.014	0.665	0.017	1.156	0.027	1.019	0.020
Ile	ATA	0.761	0.015	0.887	0.017	0.823	0.014	0.924	0.016	0.653	0.023	0.765	0.017
Leu	TTA	1.251	0.021	1.286	0.022	1.285	0.024	1.211	0.025	0.580	0.023	0.806	0.019
Leu	TTG	1.647	0.021	1.347	0.022	1.037	0.017	1.020	0.020	1.151	0.029	1.098	0.021
Leu	CTT	1.344	0.018	1.275	0.022	1.807	0.026	1.461	0.022	1.017	0.030	1.012	0.019
Leu	CTC	0.463	0.013	0.680	0.019	0.709	0.019	0.856	0.023	1.207	0.034	1.061	0.020
Leu	CTA	0.472	0.012	0.547	0.014	0.550	0.013	0.662	0.017	0.575	0.026	0.639	0.016
Leu	CTG	0.822	0.016	0.844	0.020	0.613	0.016	0.791	0.021	1.471	0.033	1.383	0.025
Lys	AAA	1.214	0.011	1.247	0.013	1.206	0.010	1.186	0.013	0.947	0.018	1.047	0.015
Lys	AAG	0.786	0.011	0.726	0.013	0.794	0.010	0.797	0.013	1.042	0.018	0.931	0.014
Phe	TTT	1.290	0.014	1.227	0.015	1.304	0.014	1.186	0.017	0.868	0.021	0.944	0.015
Phe	TTC	0.695	0.014	0.711	0.014	0.696	0.014	0.775	0.017	1.102	0.021	1.011	0.015
Pro	CCT	1.746	0.021	1.587	0.028	1.897	0.026	1.703	0.027	1.443	0.033	1.279	0.024
Pro	CCC	0.456	0.014	0.646	0.021	0.510	0.018	0.563	0.019	0.951	0.032	0.887	0.021
Pro	CCA	1.530	0.020	1.391	0.027	1.358	0.020	1.368	0.026	1.013	0.029	1.121	0.022
Pro	CCG	0.268	0.012	0.306	0.016	0.209	0.013	0.333	0.015	0.546	0.023	0.676	0.019
Ser	TCT	1.668	0.024	1.370	0.023	1.909	0.025	1.550	0.026	1.206	0.030	1.191	0.022
Ser	TCC	0.551	0.014	0.751	0.021	0.638	0.019	0.780	0.020	0.965	0.030	0.989	0.020
Ser	TCA	1.459	0.021	1.483	0.025	1.484	0.022	1.360	0.024	1.019	0.031	0.981	0.019
Ser	TCG	0.345	0.014	0.353	0.014	0.205	0.011	0.332	0.015	0.801	0.029	0.711	0.018
Ser	AGT	1.389	0.022	1.364	0.026	1.129	0.022	1.210	0.024	0.951	0.027	1.030	0.020
Ser	AGC	0.589	0.017	0.679	0.019	0.635	0.021	0.766	0.020	1.058	0.033	1.099	0.022
Thr	ACT	1.560	0.020	1.397	0.023	1.811	0.021	1.567	0.027	1.249	0.029	1.215	0.022
Thr	ACC	0.560	0.013	0.665	0.018	0.623	0.017	0.745	0.020	1.052	0.028	0.938	0.020
Thr	ACA	1.554	0.020	1.514	0.023	1.355	0.020	1.388	0.024	0.957	0.026	1.048	0.021
Thr	ACG	0.311	0.014	0.425	0.016	0.186	0.009	0.267	0.013	0.730	0.025	0.783	0.018
Tyr	TAT	1.272	0.015	1.190	0.018	1.289	0.016	1.113	0.017	0.761	0.022	0.867	0.017
Tyr	TAC	0.706	0.015	0.644	0.016	0.685	0.015	0.826	0.017	1.121	0.024	1.035	0.017

Val	GTT	1.581	0.018	1.338	0.022	1.734	0.020	1.472	0.026	1.077	0.025	1.109	0.019
Val	GTC	0.403	0.011	0.544	0.015	0.632	0.013	0.733	0.020	0.957	0.024	0.876	0.018
Val	GTA	0.849	0.014	0.975	0.020	0.875	0.016	0.983	0.022	0.697	0.023	0.818	0.018
Val	GTG	1.167	0.015	1.129	0.021	0.758	0.016	0.800	0.019	1.257	0.026	1.187	0.021
