# Supplementary material for: Codon and Amino Acid Usage Are Shaped by Selection Across Divergent Model Organisms of the Pancrustacea
Source: G3 (Bethesda). 2015 Sep 17;5(11):2307–21. doi: 10.1534/g3.115.021402 (PMC4632051; doi:10.1534/g3.115.021402)
Supplement: Supporting Information [file supp_g3.115.021402_TableS3.pdf]

Table S3 The size complexity (S/C) scores per amino acid as per Dufton et al. (1997).

Ala	A	4.76
Arg	R	56.34
Asn	N	33.72
Asp	D	32.72
Cys	C	57.16
Gln	Q	37.48
Glu	E	36.48
Gly	G	1
His	H	58.7
Ile	I	16.04
Leu	L	16.04
Lys	K	30.14
Met	M	64.68
Phe	F	44
Pro	P	31.8
Ser	S	17.86
Thr	T	21.62
Trp	W	73
Tyr	Y	57
Val	V	12.28
