# Supplementary material for: Codon and Amino Acid Usage Are Shaped by Selection Across Divergent Model Organisms of the Pancrustacea
Source: G3 (Bethesda). 2015 Sep 17;5(11):2307–21. doi: 10.1534/g3.115.021402 (PMC4632051; doi:10.1534/g3.115.021402)
Supplement: Supporting Information [file supp_g3.115.021402_TableS4.pdf]

Table S4 Functional clustering of the pooled moderate and low expressed CDS (all CDS below the 95th percentile of RPM) for each of three arthropod species under study using their orthologs in the model *D. melanogaster*. The orthologs of CDS below the 95th percentile in expression per species were submitted to gene ontology system DAVID (DUFTON 1997) using identifiers of their *D. melanogaster* orthologs. Functional categories with enrichment values >2.5 are shown. P-values represent a modified Fisher's test, wherein lower values indicate greater enrichment.

Gryllus bimaculatus		***Oncopeltus fasciatus***		***Parhyale hawaiensis***	
**Enrichment Score: 22.76**	P-Value	**Enrichment Score: 17.93**	P-Value	**Enrichment Score: 22.76**	P-value
nucleotide-binding	1.00E-27	nucleotide binding	1.30E-21	nucleotide-binding	1.90E-16
atp-binding	2.90E-27	nucleoside binding	1.50E-19	atp-binding	4.10E-14
purine ribonucleotide binding	2.80E-24	purine nucleotide binding	2.50E-19	purine ribonucleotide binding	1.30E-12
ribonucleotide binding	2.80E-24	purine nucleoside binding	5.70E-19	ribonucleotide binding	8.10E-12
purine nucleotide binding	4.00E-24	adenyl nucleotide binding	3.20E-18	purine nucleotide binding	2.10E-11
nucleoside binding	6.10E-23	ribonucleotide binding	5.40E-18	nucleoside binding	2.10E-11
purine nucleoside binding	6.30E-22	purine ribonucleotide binding	5.40E-18	purine nucleoside binding	1.50E-10
adenyl ribonucleotide binding	1.30E-21	adenyl ribonucleotide binding	3.50E-17	adenyl ribonucleotide binding	2.20E-10
nucleotide binding	1.30E-21	ATP binding	4.70E-17	nucleotide binding	4.20E-10
adenyl nucleotide binding	2.10E-21	**Enrichment Score: 15.66**		adenyl nucleotide binding	5.00E-09
ATP binding	3.90E-21	organelle lumen	2.00E-16	ATP binding	7.20E-09
**Enrichment Score: 8.93**		intracellular organelle lumen	2.00E-16	**Enrichment Score: 8.93**	
endocytosis	2.40E-10	membrane-enclosed lumen	2.80E-16	endocytosis	2.40E-07
membrane invagination	2.40E-10	**Enrichment Score: 12.68**		membrane invagination	6.00E-06
membrane organization	2.60E-08	membrane organization	8.10E-14	membrane organization	9.80E-06
**Enrichment Score: 8.51**		endocytosis	3.40E-13	**Enrichment Score: 8.51**	5.20E-04

intracellular organelle lumen	3.00E-09	membrane invagination	3.40E-13	intracellular organelle lumen	
organelle lumen	3.00E-09	**Enrichment Score: 7.58**		organelle lumen	4.50E-06
membrane-enclosed lumen	3.30E-09	protein complex biogenesis	2.20E-08	membrane-enclosed lumen	4.50E-06
**Enrichment Score: 7.29**		protein complex assembly	2.20E-08	**Enrichment Score: 7.29**	7.00E-06
serine/threonine-protein kinase	1.20E-10	macromolecular complex assembly	3.60E-08	serine/threonine-protein kinase	1.00E-04
protein amino acid phosphorylation	3.90E-09	**Enrichment Score: 7.5**		protein amino acid phosphorylation	1.90E-04
protein kinase activity	1.80E-08	cellular macromolecule catabolic process	3.60E-10	protein kinase activity	3.20E-04
Protein kinase	4.5E-05	macromolecule catabolic process	4.70E-09	Protein kinase	3.90E-04
protein serine/threonine kinase activity	2.00E-08	protein catabolic process	7.50E-08	protein serine/threonine kinase activity	4.90E-04
Protein kinase	5.5E-02	modification-dependent protein catabolic process	9.90E-08	Protein kinase	6.00E-04
Serine/threonine protein kinase	5.5E-02	modification-dependent macromolecule catabolic process	1.30E-07	Serine/threonine protein kinase	
Serine/threonine protein kinase-related	4.40E-06	proteolysis involved in cellular protein catabolic process	1.40E-07	Serine/threonine protein kinase-related	2.20E-08
**Enrichment Score: 6.76**		cellular protein catabolic process	1.40E-07	**Enrichment Score: 6.76**	2.30E-06
phosphorylation	1.70E-07	**Enrichment Score: 6.49**		phosphorylation	3.10E-06
phosphorus metabolic process	1.70E-07	helicase	4.50E-09	phosphorus metabolic process	1.10E-05
phosphate metabolic process	1.70E-07	purine NTP-dependent helicase activity	8.30E-08	phosphate metabolic process	1.10E-05
**Enrichment Score: 6.59**		ATP-dependent helicase activity	8.30E-08	**Enrichment Score: 6.59**	2.00E-04
wd repeat	1.50E-09	DEXDc	1.80E-07	wd repeat	1.00E-03
WD40	2.30E-07	HELICc	2.80E-07	WD40	3.50E-03
WD40/YVTN repeat-like	2.50E-07	DEAD-like helicase	1.60E-06	WD40/YVTN repeat-like	4.30E-03
WD40 repeat	3.80E-07	DNA/RNA helicase	2.50E-06	WD40 repeat	5.70E-03

WD40 repeat		Helicase	2.50E -06	WD40 repeat	2.10E-02
WD40 repeat	2.40E-03	**Enrichment Score: 6.06**		**WD40 repeat**	
WD40 repeat 2	2.90E-06	organellar ribosome	9.20E-09	WD40 repeat 2	1.00E-04
**Enrichment Score: 6.31**		mitochondrial ribosome	9.20E-09	**Enrichment Score: 6.31**	1.00E-04
helicase	3.30E-08	ribosomal subunit	7.80E-03	helicase	2.20E-04
HELICc	4.20E-07	**Enrichment Score: 5.96**		HELICc	6.70E-04
DNA/RNA helicase	1.40E-01	phosphorus metabolic process	1.00E-06	**DNA/RNA helicase**	
DEXDc	9.10E-07	phosphate metabolic process	1.00E-06	DEXDc	1.30E-05
DEAD-like helicase	1.80E-01	phosphorylation	1.30E-06	DEAD-like helicase	1.40E-05
Helicase	2.50E-01	**Enrichment Score: 5.9**		Helicase	8.00E-05
**Enrichment Score: 5.79**		cellular protein localization	6.00E-07	**Enrichment Score: 5.79**	1.30E-04
cellular macromolecule catabolic process	2.90E-08	cellular macromolecule localization	1.80E-06	cellular macromolecule catabolic process	1.30E-04
protein catabolic process	3.20E-07	intracellular protein transport	1.90E-06	protein catabolic process	1.70E-04
modification-dependent protein catabolic process	4.50E-06	**Enrichment Score: 5.85**		modification-dependent protein catabolic process	1.90E-04
modification-dependent macromolecule catabolic process	5.60E-06	transcription initiation from RNA polymerase II promoter	3.50E-07	modification-dependent macromolecule catabolic process	2.90E-04
proteolysis involved in cellular protein catabolic process	8.80E-06	general RNA polymerase II transcription factor activity	2.50E-06	proteolysis involved in cellular protein catabolic process	1.30E-01
cellular protein catabolic process	8.80E-06	transcription initiation	3.30E-06	cellular protein catabolic process	7.40E-01
**Enrichment Score: 5.71**		**Enrichment Score: 5.18**		**Enrichment Score: 5.71**	
Tetratricopeptide TPR-1	1.50E-07	PHD	1.70E -07	Tetratricopeptide TPR-1	1.10E-04
TPR	3.20E-06	Zinc finger	9.50E-07	TPR	1.10E-04
Tetratricopeptide repeat	3.80E-06	Zinc finger	4.60E-05	Tetratricopeptide repeat	1.50E-04
Tetratricopeptide region	7.60E-06	Zinc finger		Tetratricopeptide region	3.30E-04

**Enrichment Score: 5.45**		**Enrichment Score: 4.75**		**Enrichment Score: 5.45**	2.80E-03
RNA transport	6.60E-07	DNA-directed RNA polymerase activity	1.00E-06	RNA transport	4.30E-03
nucleic acid transport	6.60E-07	RNA polymerase activity	1.00E-06	**nucleic acid transport**	
establishment of RNA localization	1.00E-06	RNA polymerase	5.60E-03	establishment of RNA localization	6.00E-06
nucleobase	1.90E-01	**Enrichment Score: 4.74**		nucleobase	1.30E-04
RNA localization	7.70E-04	ubiquitin-protein ligase activity	6.50E-06	RNA localization	2.00E-04
**Enrichment Score: 5.01**		small conjugating protein ligase activity	7.10E-06	**Enrichment Score: 5.01**	2.70E-03
nucleoside-triphosphatase regulator activity	4.40E-06	acid-amino acid ligase activity	1.30E-04	nucleoside-triphosphatase regulator activity	3.10E-03
GTPase regulator activity	4.80E-06	**Enrichment Score: 4.39**		GTPase regulator activity	3.50E-03
small GTPase regulator activity	4.50E-05	mitochondrial large ribosomal subunit	1.90E-06	small GTPase regulator activity	8.30E-03
**Enrichment Score: 4.11**		organellar large ribosomal subunit	1.90E-06	**Enrichment Score: 4.11**	
PHD	3.30E-05	large ribosomal subunit	2.00E-02	PHD	2.80E-05
Zinc finger	5.30E-01	**Enrichment Score: 4.25**		Zinc finger	1.50E-03
Zinc finger	8.00E-01	maintenance of protein location	1.20E-05	Zinc finger	4.30E-02
Zinc finger		maintenance of location	3.10E-05	**Zinc finger**	
**Enrichment Score: 4.07**		maintenance of protein location in cell	1.00E-04	**Enrichment Score: 4.07**	4.30E-05
cell-cell junction organization	1.60E-05	maintenance of location in cell	2.70E-04	cell-cell junction organization	2.10E-04
cell junction organization	2.80E-05	**Enrichment Score: 4.11**		cell junction organization	1.20E-02
apical junction assembly	9.10E-05	cation binding	3.60E-05	apical junction assembly	6.80E-02
cell-cell junction assembly	2.60E-04	metal ion binding	3.60E-05	cell-cell junction assembly	
cell junction assembly	4.30E-04	ion binding	6.00E-05	cell junction assembly	7.60E-05
**Enrichment Score: 3.86**		transition metal ion binding	4.40E-04	**Enrichment Score: 3.86**	4.70E-04

DNA-directed RNA polymerase complex	6.50E-05	**Enrichment Score: 4.05**		DNA-directed RNA polymerase complex	1.10E-03
nuclear DNA-directed RNA polymerase complex	6.50E-05	protein amino acid phosphorylation	8.30E-06	nuclear DNA-directed RNA polymerase complex	2.40E-03
RNA polymerase complex	6.50E-05	Protein kinase	2.70E-04	RNA polymerase complex	3.90E-03
RNA polymerase activity	1.70E-04	protein kinase activity	3.10E-04	RNA polymerase activity	5.20E-03
DNA-directed RNA polymerase activity	1.70E-04	**Enrichment Score: 3.85**		DNA-directed RNA polymerase activity	6.90E-03
RNA polymerase	8.20E-04	generation of a signal involved in cell-cell signaling	2.30E-05	RNA polymerase	2.90E-02
**Enrichment Score: 3.36**		secretion by cell	4.10E-05	**Enrichment Score: 3.36**	
aging	4.30E-04	neurotransmitter secretion	4.20E-05	aging	2.50E-07
determination of adult life span	4.30E-04	regulation of neurotransmitter levels	1.40E-04	determination of adult life span	3.10E-05
multicellular organismal aging	4.30E-04	secretion	2.10E-04	multicellular organismal aging	1.10E-04
Enrichment Score: 3.32		neurotransmitter transport	6.40E-03	**Enrichment Score: 3.32**	1.30E-04
sh3 domain	7.50E-05	**Enrichment Score: 3.7**		sh3 domain	4.80E-04
SH3	1.10E-03	kelch repeat	6.90E-05	SH3	1.00E-03
Src homology-3 domain	1.30E-03	Kelch	1.70E -04	Src homology-3 domain	1.30E-03
**Enrichment Score: 3.24**		Kelch-type beta propeller	3.70E-04	Enrichment Score: 3.24	3.10E-03
kelch repeat	2.60E-04	Kelch repeat type 1	3.70E-04	kelch repeat	1.20E-02
Kelch	7.20E-04	**Enrichment Score: 3.66**		Kelch	8.60E-02
Kelch repeat type 1	7.70E-04	wd repeat	4.60E-07	Kelch repeat type 1	1.10E-01
Kelch-type beta propeller	7.70E-04	WD40	6.10E -05	Kelch-type beta propeller	1.70E-01
**Enrichment Score: 3.21**		WD40/YVTN repeat-like	1.90E-04	Enrichment Score: 3.21	1.80E-01
gtp-binding	2.70E-04	WD40 repeat	6.30E-04	gtp-binding	1.90E-01
guanyl ribonucleotide binding	7.40E-04	WD40 repeat	7.90E-04	guanyl ribonucleotide binding	

GTP binding	8.20E-04	WD40 repeat	1.70E-03	GTP binding	2.50E-05
guanyl nucleotide binding	8.70E-04	WD40 repeat 2	5.40E-03	guanyl nucleotide binding	4.30E-05
**Enrichment Score: 3.1**		**Enrichment Score: 3.65**		**Enrichment Score: 3.1**	8.00E-04
ank repeat	1.80E-04	ank repeat	4.20E-05	ank repeat	3.70E-03
ANK	1.50E-03	ANK	2.30E -04	ANK	1.00E-02
Ankyrin	1.80E-03	Ankyrin	1.10E -03	Ankyrin	1.50E-02
**Enrichment Score: 3.07**		**Enrichment Score: 3.64**		**Enrichment Score: 3.07**	3.00E-02
epithelium development	3.40E-04	nuclear DNA-directed RNA polymerase complex	7.80E-05	epithelium development	RT
morphogenesis of an epithelium	7.40E-04	DNA-directed RNA polymerase complex	7.80E-05	morphogenesis of an epithelium	5.20E-02
tissue morphogenesis	2.50E-03	RNA polymerase complex	7.80E-05	tissue morphogenesis	1.70E-01
**Enrichment Score: 2.83**		RNA polymerase	5.60E-03	**Enrichment Score: 2.83**	
glycerophospholipid metabolic process	3.00E-04	**Enrichment Score: 3.25**		glycerophospholipid metabolic process	8.70E-06
glycerolipid metabolic process	6.70E-04	serine/threonine-protein kinase	1.00E-04	glycerolipid metabolic process	8.50E-05
phospholipid metabolic process	3.00E-03	Serine/threonine protein kinase-related	4.50E-04	phospholipid metabolic process	5.90E-04
organophosphate metabolic process	8.20E-03	protein serine/threonine kinase activity	8.10E-04	organophosphate metabolic process	5.90E-04
**Enrichment Score: 2.71**		Serine/threonine protein kinase	2.80E-03	**Enrichment Score: 2.71**	6.00E-04
RNA-dependent ATPase activity	1.50E-03	**Enrichment Score: 3.22**		RNA-dependent ATPase activity	7.40E-04
ATP-dependent RNA helicase activity	1.50E-03	ribosomal protein	3.40E-06	ATP-dependent RNA helicase activity	2.90E-03
RNA helicase activity	3.40E-03	structural constituent of ribosome	1.20E-03	RNA helicase activity	3.40E-03
**Enrichment Score: 2.62**		ribosome	4.30E-03	**Enrichment Score: 2.62**	
ubiquitin-protein ligase activity	3.50E-04	ribosomal subunit	7.80E-03	ubiquitin-protein ligase activity	6.30E-03
small conjugating protein ligase activity	1.50E-03	**Enrichment Score: 3.22**		small conjugating protein ligase activity	1.50E-02

ligase activity	5.40E-02	aging	6.00E -04	ligase activity	1.60E-02
acid-amino acid ligase activity	1.00E-02	determination of adult life span	6.00E-04	acid-amino acid ligase activity	1.60E-02
**Enrichment Score: 2.59**		multicellular organismal aging	6.00E-04	**Enrichment Score: 2.59**	RT
metal ion binding	1.70E-03	**Enrichment Score: 3.16**		metal ion binding	2.50E-02
cation binding	2.40E-03	transmission of nerve impulse	5.50E-04	cation binding	9.80E-01
ion binding	2.90E-03	synaptic transmission	5.60E-04	ion binding	4.20E-01
transition metal ion binding	3.80E-03	cell-cell signaling	1.00E-03	transition metal ion binding	5.30E-01
**Enrichment Score: 2.53**		**Enrichment Score: 3.14**		**Enrichment Score: 2.53**	
mitochondrial small ribosomal subunit	3.50E-04	glycerophospholipid metabolic process	1.70E-04	mitochondrial small ribosomal subunit	2.20E-08
organellar small ribosomal subunit	3.50E-04	glycerolipid metabolic process	4.60E-04	organellar small ribosomal subunit	2.60E-05
small ribosomal subunit	2.00E-01	phosphoinositide metabolic process	5.00E-03	small ribosomal subunit	6.50E-05
		**Enrichment Score: 2.86**			
		nucleoside-triphosphatase regulator activity	7.50E-04		
		GTPase regulator activity	1.50E-03		
		small GTPase regulator activity	2.40E-03		
		**Enrichment Score: 2.83**			
		regulation of actin polymerization or depolymerization	8.60E-04		
		regulation of actin filament length	8.60E-04		
		regulation of actin filament polymerization	4.50E-03		
		**Enrichment Score: 2.78**			
		apicolateral plasma membrane	3.90E-04		
		apical junction complex	1.90E-03		

cell-cell junction	6.10E-03
**Enrichment Score: 2.72**	
RNA helicase activity	5.20E-04
ATP-dependent RNA helicase activity	3.70E-03
RNA-dependent ATPase activity	3.70E-03
**Enrichment Score: 2.64**	
mitochondrial electron transport	6.10E-04
oxidoreductase activity	7.50E-04
NADH dehydrogenase activity	1.40E-03
NADH dehydrogenase (quinone) activity	2.50E-03
oxidoreductase activity NADH dehydrogenase (ubiquinone) activity	2.50E-03
NADH dehydrogenase complex	5.40E-03
respiratory chain complex I	5.40E-03
mitochondrial respiratory chain complex I	5.40E-03
Enrichment Score: 2.59	
establishment of RNA localization	1.50E-03
RNA transport	2.70E-03
nucleic acid transport	2.70E-03
nucleobase	
**Enrichment Score: 2.56**	
apical junction assembly	1.20E-03

cell-cell junction assembly	2.80E-03
cell-cell junction organization	2.90E-03
cell junction organization	4.10E-03
cell junction assembly	4.20E-03
**Enrichment Score: 2.55**	
organellar small ribosomal subunit	4.70E-04
mitochondrial small ribosomal subunit	4.70E-04
small ribosomal subunit	1.00E-01
**Enrichment Score: 2.53**	
Spectrin repeat	1.80E-03
SPEC	2.90E-03
Spectrin/alpha-actinin	4.70E-03
**Enrichment Score: 2.53**	
nuclear division	2.30E-03
organelle fission	2.90E-03
mitosis	2.90E-03
M phase of mitotic cell cycle	4.00E-03
**Enrichment Score: 2.5**	
3'-5'-exoribonuclease activity	2.40E-03
exoribonuclease activity	3.60E-03
exoribonuclease activity	3.60E-03
