# Supplementary material for: Codon and Amino Acid Usage Are Shaped by Selection Across Divergent Model Organisms of the Pancrustacea
Source: G3 (Bethesda). 2015 Sep 17;5(11):2307–21. doi: 10.1534/g3.115.021402 (PMC4632051; doi:10.1534/g3.115.021402)
Supplement: Supporting Information [file supp_g3.115.021402_FigureS1.pdf]

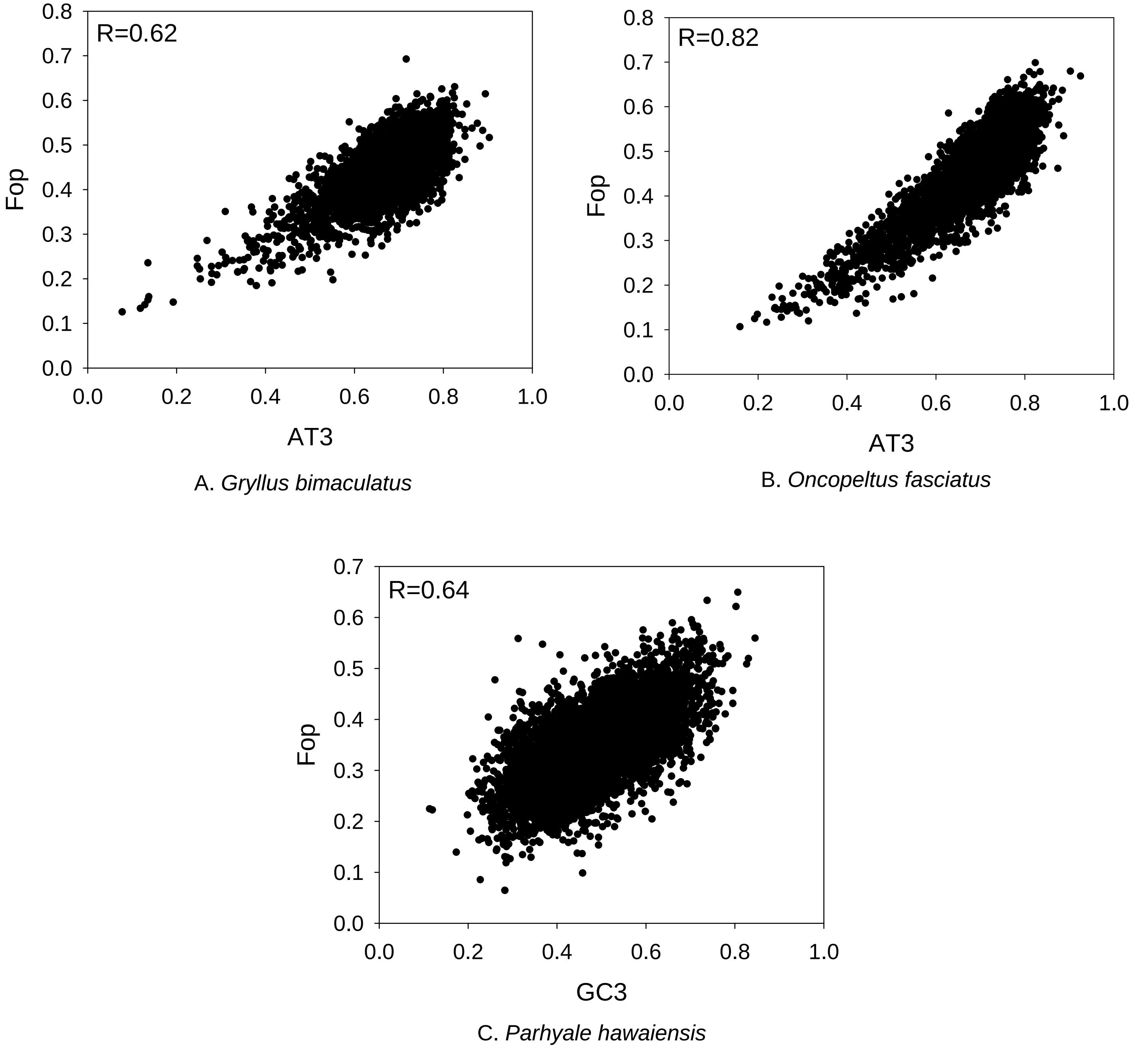

Figure S1 The Spearman rank correlation A) AT3 and Fop for *G. bimaculatus*. B) AT3 and Fop for *O. fasciatus*. C) GC3 and Fop for *P. hawaiensis*. P< 10^{-15} for all correlations. Pearson correlations yielded nearly identical results (not shown).
