# Supplementary material for: Codon and Amino Acid Usage Are Shaped by Selection Across Divergent Model Organisms of the Pancrustacea
Source: G3 (Bethesda). 2015 Sep 17;5(11):2307–21. doi: 10.1534/g3.115.021402 (PMC4632051; doi:10.1534/g3.115.021402)
Supplement: Supporting Information [file supp_g3.115.021402_FigureS2.pdf]

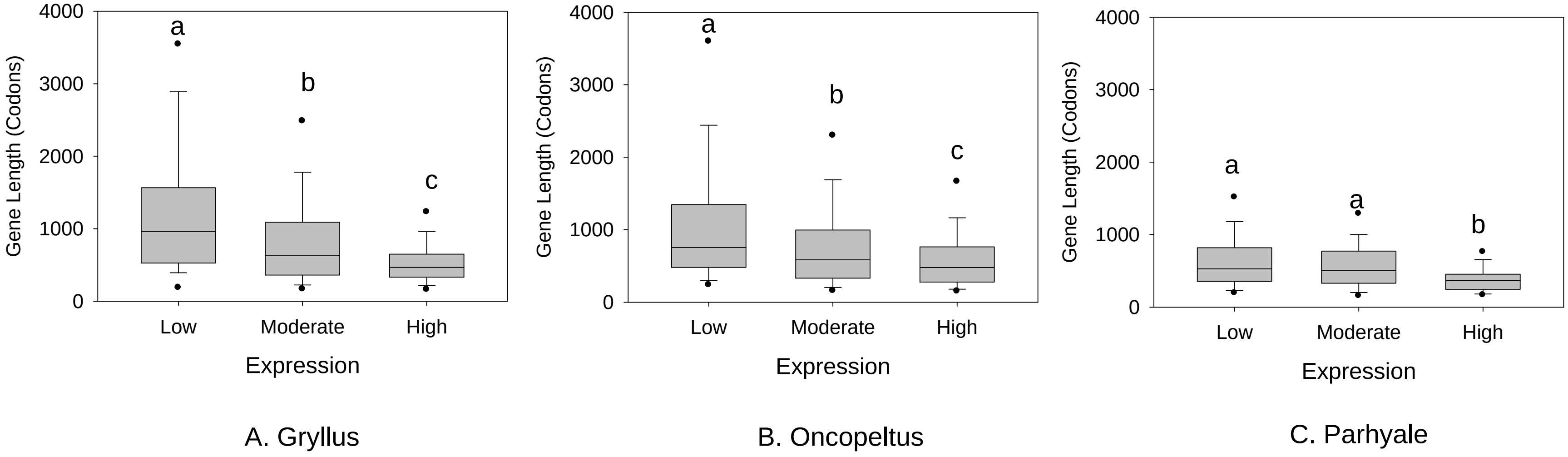

Figure S2 Bar and whisker plots of CDS length (number of codons) of *D. melanogaste*r orthologs to CDS with low, moderate and high expression in A) *G. bimaculatus;* B) *O. fasciatus*; and C) *P. hawaiensis*. P-values of Ranked-ANOVA $<3.9X10^{-9}$ for each figure. Different letters in each figure indicate paired differences using Dunn's contrast (P<0.05).
