# Supplementary material for: Codon and Amino Acid Usage Are Shaped by Selection Across Divergent Model Organisms of the Pancrustacea
Source: G3 (Bethesda). 2015 Sep 17;5(11):2307–21. doi: 10.1534/g3.115.021402 (PMC4632051; doi:10.1534/g3.115.021402)
Supplement: Supporting Information [file supp_g3.115.021402_FileS1.pdf]

File S1

Supporting Results File 1

In the analysis of RSCU described in our Results section, we chose to use RPM to measure expression rather than reads per kilobase million (RPKM). This is because while the CDS lengths of assembled transcripts for *G. bimaculatus* and *O. fasciatus* were generated using both normalized and non-normalized libraries (EWEN-CAMPEN *et al.* 2011; ZENG *et al.* 2013; ZENG and EXTAVOUR 2012), we quantified expression levels for this study using solely non-normalized libraries, which most directly correlates to expression level (OSHLACK *et al.* 2010). We anticipated that using RPKM rather than RPM would thus likely skew some highly expressed genes towards lower values by underestimating their expression levels, because a transcript segment present in normalized libraries may contribute to transcript assembly by extending gene length, but not have hits in the non-normalized dataset. Indeed, this prediction was borne out when we determined optimal codons using RPKM. Overall we obtained similar results to those obtained with the RPM method (Table 1). However, for *G. bimaculatus* we identified only 10 of the original 17 optimal codons, as seven became non-significant (importantly, 17 of the 17 had positive ΔRSCU using RPM and RPKM, consistent with optimization detected using both methods), whilst we identified 12 of the original 16 optimal codons for *O. fasciatus* (16 of the 16 optimal codons had positive ΔRSCU using RPM and RPKM) (Table 1). For *P. hawaiensis*, we found the exact same 13 optimal codons as those originally identified with the RPM method (Table 1), consistent with the fact that normalized libraries were not used for the transcriptome assembly in this species (ZENG *et al.* 2011). Thus, there is moderate variation in P-values among results obtained with the RPM and RPKM methods. Collectively, from these data we conclude that the only potential effect of using RPM (as opposed to RPKM) to define our 5% most highly or lowly expressed gene lists would possibly be an over-representation of highly expressed long CDS (relative to highly expressed shorter CDS), due to more read matches to longer CDS. This could only affect our results if both the following were true: 1) longer CDS exhibited elevated levels of AT3 or GC3 for reasons other than selection on codon usage (e.g. mutational bias), and 2) the high expression dataset consisted mostly of long genes. However, we examined these possibilities empirically and found that neither of these factors play a role here. To test mutation, we examined the lowest expression 5% RPM category, where selection effects on codon usage should be minimal or

absent (and thus AT variation should be explained solely by mutation): we found no correlation between CDS length (that predicted using transcript read assembly) and AT3 content for *G. bimaculatus* (Spearmans Correlation P=0.37) or *O. fasciatus* (P=0.85), indicating no evidence of a relationship between mutational bias and assembled CDS length. In terms of CDS length, we found the CDS sequences assembled in the 5% highest RPM class consisted of a range of short and long lengths (for example, for *G. bimaculatus* CDS ranged between 102 codons to 2039, with a mean of 466±16.7), and thus spans a range of lengths. Taken together, we conclude the RPM values (as compared to RPKM) provide the most rigorous method for identification of optimal codons in these datasets, as indicated by a strong correspondence to results from RPKM, but with stronger P-values.

It is worth noting that our results showing Fop increases with expression level in Figure 2 were the same regardless of whether we used the RPM or RPKM list of optimal codons. For instance, using the RPKM list for *G. bimaculatus* and for *O. fasciatus*, Fop was found to increase from the low ($Mean_{G.\ bimaculatus}$=0.352±0.006, $Mean_{O.\ fasciatus}$,=0.336±0.005), to the moderate $Mean_{G.\ bimaculatus}$ 0.374±0.001, $Mean_{O.\ fasciatus}$,=0.373±0.001) to the high expression ($Mean_{G.\ bimaculatus}$=0.403±0.003, $Mean_{O.\ fasciatus}$,=0.391±0.004) class for *G. bimaculatus* and for *O. fasciatus* (Ranked ANOVA P<0.001, Dunns Paired test P<0.05 for each contrast per species).
