# Supplementary material for: Codon and Amino Acid Usage Are Shaped by Selection Across Divergent Model Organisms of the Pancrustacea
Source: G3 (Bethesda). 2015 Sep 17;5(11):2307–21. doi: 10.1534/g3.115.021402 (PMC4632051; doi:10.1534/g3.115.021402)
Supplement: Supporting Information [file supp_g3.115.021402_FileS2.pdf]

File S2

Supporting Results File 2

We identified orthologs of the 87 ribosomal protein genes (RPGs) in *D. melanogaster* (http://ribosome.med.miyazaki-u.ac.jp/) using BLASTX to the reduced CDS list (without isoforms, and with ORF with a start codon) in *G. bimaculatus*, *O. fasciatus*, and of *P. hawaiensis*. We then concatenated CDS for the RPGs dataset and for the lowly expressed CDS per species, and determined $\Delta RSCU_{RPGs} = RSCU_{RPGs} - RSCU_{CDS\ with\ Lowest\ 5\%\ Expression}$. Whilst signals were weakened as compared to the full high expression gene set used in Table 1, especially for two-fold synonymous sites in *G. bimaculatus* and *O. fasciatus* (likely due to the small dataset size of RPGs and low selection at two-fold sites (Table 1)), the results from amino acids with three or more amino acids that exhibit the greatest selection on codon usage (Table 1), support the presence of AT3 optimal codons in these organisms. For instance, for *G. bimaculatus* and for *O. fasciatus*, the optimal codon for nearly all of nine amino acids having three or more synonymous codons in Table 1, yielded a positive $\Delta RSCU_{RPGs}$ (values between +0.12 and +0.73), thus confirming their enhanced usage in highly expressed genes (RPGs). An exception was Arg in *O. fasciatus*, where the optimal codon identified in Table 1 was CGT, even though AGG had a larger ΔRSCU (non-significant); using RPGs, AGG had fourfold higher $\Delta RSCU_{RPGs}$,. A second exception was Pro for *G. bimaculatus* where the optimal codon using RPGs was CCT rather than CCA. For *P. hawaiensis*, 11 of the 13 optimal codons in Table 1 were also identified using $\Delta RSCU_{RPGs}$,. A switch was observed for two amino acids: GGA to GGT for Gly and TCG to TCC for Ser, each staying within the AT3 or GC3 codon family, respectively. Notably, additional amino acids had codons with substantial positive $\Delta RSCU_{RPGs}$ for *P. hawaiensis* and might be putative optimal codons, such as Arg (both CGC and CGT), Cys (TGC), His (CAC) and Glu (GAG). Thus, GC3 codons might be favored across a wider spectrum of amino acids than reported in Table 1 ($\Delta RSCU_{RPGs}$ values ranged from +0.20 to +0.73). We therefore consider the lists in Table 1 for *P. hawaiensis* spanning 13 amino acids to be conservative. Future genomic sequence data will help resolve these variations. Together, $\Delta RSCU_{RPGs}$ analysis concurs with prevalence of AT3 optimal codons in *G. bimaculatus* and *O. fasciatus*, and GC3 optimal codons in *P. hawaiensis*.
