# Supplementary material for: Codon and Amino Acid Usage Are Shaped by Selection Across Divergent Model Organisms of the Pancrustacea
Source: G3 (Bethesda). 2015 Sep 17;5(11):2307–21. doi: 10.1534/g3.115.021402 (PMC4632051; doi:10.1534/g3.115.021402)
Supplement: Supporting Information [file supp_g3.115.021402_FileS3.pdf]

File S3
Supporting Materials and Methods

Transcriptome data from RNA-seq for oogenesis and embryogenesis of *G. bimaculatus* (ZENG *et al.* 2013) *O. fasciatus* (EWEN-CAMPEN *et al.* 2011) and *P. hawaiensis* (ZENG *et al.* 2011) were obtained from ASGARD as shown in supplementary Table S1. For each species, we divided the CDS list into two categories: those with isoforms and those without isoforms. The latter class was used for our analyses and to map reads; this allows certainty when matching reads, as isoforms from a single gene can match the same read. In turn, for this reduced CDS set with no isoforms, we extracted the open reading frame (ORF) using ORF Predictor (http://proteomics.ysu.edu/tools/OrfPredictor.html). The final CDS set per species was used to study gene expression profiles, and to identify the sets of the 5% of most highly and lowly expressed genes, which was then used to reveal their optimal codon lists.

Expression level was measured based on the number of hits per CDS for genes without isoforms using MEGABLAST. For each read, the CDS with the greatest percent identity was taken as the match, with a cutoff of >95% identity. Each read matched only one CDS. Expression levels per CDS were calculated by scoring the number of reads mapped from each non-normalized library to the CDS list per species for all genes without isoforms (supplementary Table S1), and was standardized as Reads per million (RPM) = Number of matching reads/Total number of reads matching a CDS X 1,000,000. Reads per kilobase million (RPKM) was calculated as RPM/CDS length X 1,000. Fop was determined using Codon W (Peden, http://codonw.sourceforge.net/). Ribosomal protein genes were identified using BLASTX to query the *D. melanogaster* RPG list http://ribosome.med.miyazaki-u.ac.jp/). Orthologs between *G. bimaculatus*, *O. fasciatus* and *P. hawaiensis* and *D. melanogaster* were also identified using BLASTX, with the latter taxon used as the protein sequence database using the longest CDS per gene. Gene ontology was assessed using DAVID Bioinformatics Resources 6.7 (HUANG DA *et al.* 2009a; HUANG DA *et al.* 2009b). Statistical analysis was conducted using SigmaStat 3.5 (http://www.systat.com).
